# The evolution of the Sesia Zone (Western Alps) from Carboniferous to Cretaceous: insights from zircon and allanite geochronology

**DOI:** 10.1186/s00015-020-00372-4

**Published:** 2020-12-07

**Authors:** Alice Vho, Daniela Rubatto, Pierre Lanari, Daniele Regis

**Affiliations:** 1 Institute of Geological Sciences, University of Bern, Baltzerstrasse 1+3, CH-3012 Bern, Switzerland; 2 Institut des Sciences de La Terre, University of Lausanne, CH-1015 Lausanne, Switzerland; 3 Geological Survey of Canada, Ottawa, ON K1A 0E8 Canada

**Keywords:** U-Th-Pb dating, SIMS, LA-ICP-MS, Permian metamorphism, Alpine subduction

## Abstract

Microscale dating of distinct domains in minerals that contain relics of multiple metamorphic events is a key tool to characterize the polyphase evolution of complex metamorphic terranes. Zircon and allanite from five metasediments and five metaintrusive high-pressure (HP) rocks from the Eclogite Micaschist Complex of the Sesia Zone were dated by SIMS and LA-ICP-MS. In the metasediments, zircon systematically preserves detrital cores and one or two metamorphic overgrowths. An early Permian age is obtained for the first zircon rim in metasediments from the localities of Malone Valley, Chiusella Valley and Monte Mucrone (292 ± 11, 278.8 ± 3.6 and 285.9 ± 2.9 Ma, respectively). In the Malone Valley and Monte Mucrone samples, the early Permian ages are attributed to high-temperature metamorphism and coincide with the crystallization ages of associated mafic and felsic intrusions. This implies that magmatism and metamorphism were coeval and associated to the same tectono-metamorphic extensional event. In the Malone Valley, allanite from a metasediment is dated at 241.1 ± 6.1 Ma and this age is tentatively attributed to a metasomatic/metamorphic event during Permo-Triassic extension. Outer zircon rims with a late Cretaceous age (67.4 ± 1.9 Ma) are found only in the micaschist from Monte Mucrone. In metagabbro of the Ivozio Complex, zircon cores yield an intrusive age for the protolith of 340.7 ± 6.8 Ma, whereas Alpine allanite are dated at 62.9 ± 4.2 and 55.3 ± 7.3 Ma. The Cretaceous ages constrain the timing of the HP metamorphic stage. The presence of zircon overgrowth only in the central area of the Eclogite Micaschist Complex is attributed to local factors such as (1) multiple fluid pulses at HP that locally enhanced zircon dissolution and recrystallization, and (2) slightly higher temperatures reached in this area during HP metamorphism.

## Introduction

The Sesia Zone (SZ) in the Italian Western Alps represents a slice of Adriatic continental crust that underwent blueschist to eclogite facies metamorphism during convergence between the African and European plates. Since more than 50 years, extensive structural and petrological studies have been focused on unravelling the pressure–temperature (P–T) evolution of the SZ during the subduction, exhumation and continental collision of the Alpine orogenic cycle (e.g. Dal Piaz et al. 1972; Compagnoni 1977; Pognante et al. 1980; Oberhänsli et al. 1985; Zucali et al. 2004; Regis et al. 2014; Giuntoli et al. 2018a). The SZ is however composed by large sections of pre-Alpine basement that record evolution through the Variscan collision and post-Variscan extension and rifting, before being involved in the Alpine cycle (e.g. Lardeaux and Spalla 1991; Rubatto et al. 1999; Giuntoli et al. 2018a, 2018b). Reconstructing the pre-Alpine evolution of the SZ is a necessary condition for understanding its Alpine history.

**Fig. 1:**
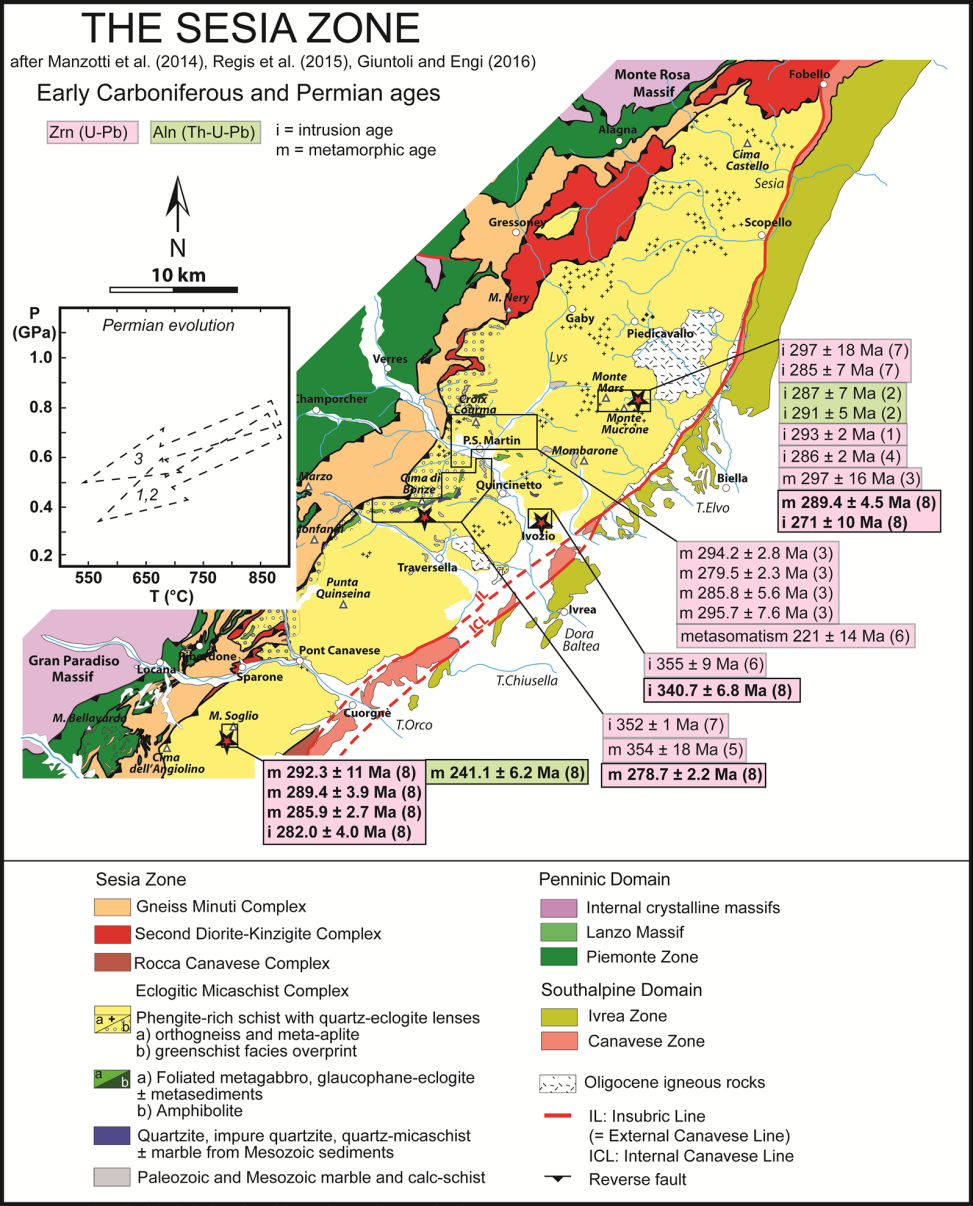
Geotectonic map of the Sesia Zone (after Manzotti et al. 2014; Regis et al. 2015; Giuntoli and Engi 2016) showing age data for the pre-Alpine metamorphism in the Eclogitic Micaschist Complex. Age data are from: (1) Bussy et al. (1998); (2) Cenki-Tok et al. (2011); (3) Kunz et al. (2018); (4) Paquette et al. (1989); (5) Regis et al. (2014); (6) Rubatto (1998); (7) Rubatto et al. (1999) and (8) this study. P–T paths for the EMC are from: (1) Lardeaux and Spalla (1991); (2) Rebay and Spalla (2001); (3) Giuntoli et al. (2018a)

Dating of robust mineral relics that may survive multiple metamorphic events is a key tool to study terranes characterized by a polymetamorphic evolution. Metamorphic conditions have been extensively determined for both the pre-Apine and Alpine evolution of the SZ (e.g. Dal Piaz et al. 1972; Compagnoni 1977; Pognante et al. 1980; Oberhänsli et al. 1985; Pognante 1989a, 1991; Lardeaux and Spalla 1991; Rebay and Spalla 2001; Zucali et al. 2002; Giuntoli et al. 2018b). Reliable age data on the pre-Alpine history remain limited (Fig. 1; Paquette et al. 1989; Bussy et al. 1998; Rubatto 1998; Rubatto et al. 1999; Cenki-Tok et al. 2011; Regis et al. 2014; Kunz et al. 2018). In particular, only a few studies have presented age data obtained by in situ analysis techniques (Cenki-Tok et al. 2011; Rubatto et al. 2011; Halama et al. 2014; Regis et al. 2014; Giuntoli et al. 2018a; Kunz et al. 2018), which are better suited to unravel different stages of complex evolutions. This represents a significant limitation considering that the SZ is composed of slices that may have different origin and P–T–time paths (Babist et al. 2006; Regis et al. 2014; Giuntoli et al. 2018b).

**Fig. 2:**
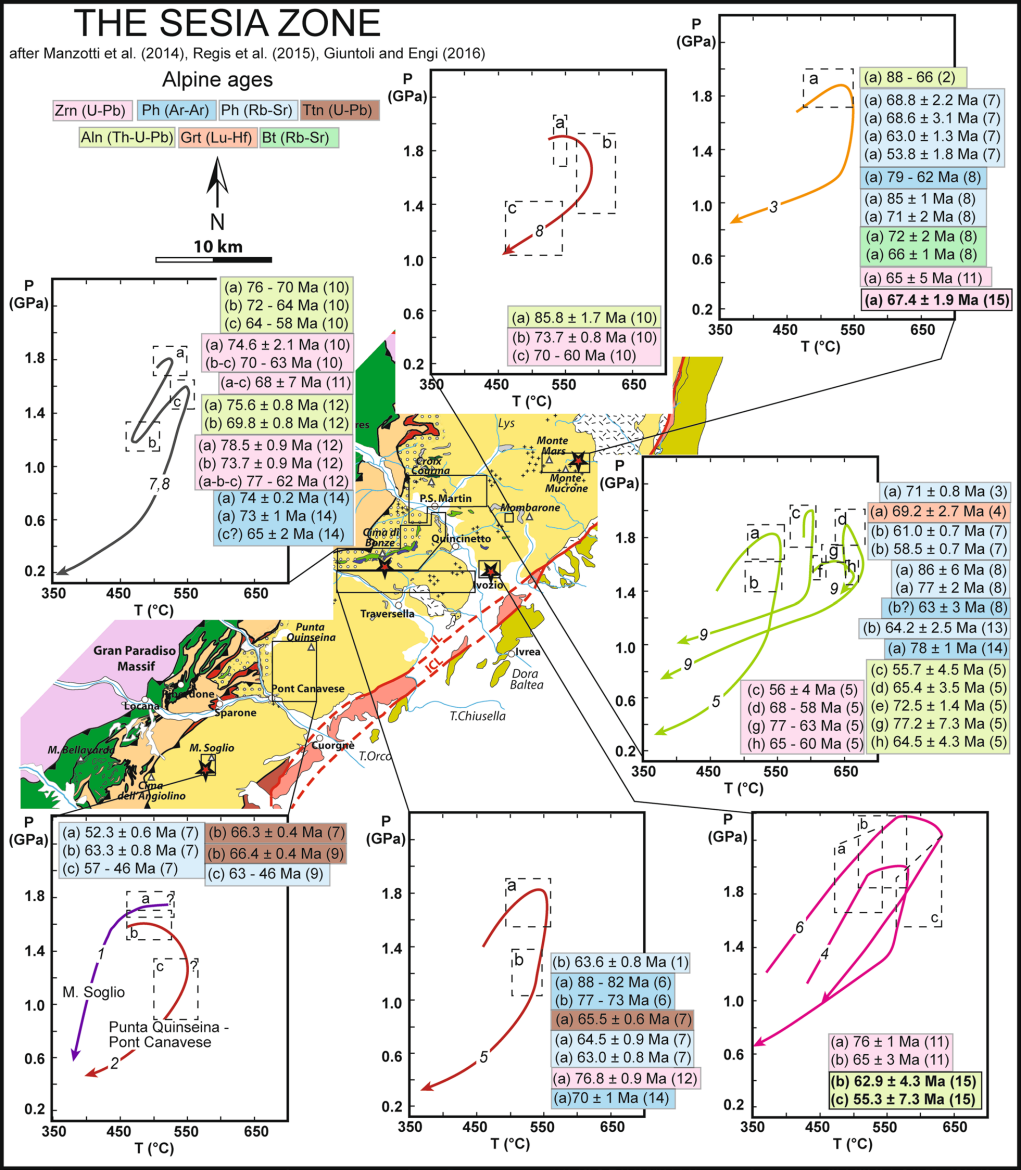
Geotectonic map of the Sesia Zone (after Manzotti et al. 2014; Regis et al. 2015; Giuntoli and Engi 2016) showing age data for the Alpine metamorphism in the Eclogitic Micaschist Complex (legend in Fig. 1). Age data are from: (1) Babist et al. (2006); (2) Cenki-Tok et al. (2011); (3) Dal Piaz et al. (2001); (4) Duchêne et al. (1997); (5) Giuntoli et al. (2018a); (6) Halama et al. (2014); (7) Inger et al. (1996); (8) Oberhänsli et al. (1985); (9) Ramsbotham et al. (1994); (10) Regis et al. (2014); (11) Rubatto et al. (1999); (12) Rubatto et al. (2011); (13) Ruffet et al. (1997); (14) Venturini (1995) and (15) this study. P–T paths are from: (1) Pognante (1989a); (2) Inger et al. (1996); (3) Zucali et al. (2002); (4) Zucali et al. (2004); (5) Babist et al. (2006); (6) Zucali and Spalla (2011); (7) Rubatto et al. (2011); (8) Regis et al. (2014); (9) Giuntoli et al. (2018a, b)

Initial efforts in determining the timing of Alpine metamorphism focused on phengite and biotite dating (Oberhänsli et al. 1985; Venturini 1995) and Rb–Sr geochronology (Oberhänsli et al. 1985; Ramsbotham et al. 1994; Venturini 1995; Inger et al. 1996; Ruffet et al. 1997; Dal Piaz et al. 2001; Babist et al. 2006) (Fig. 2). However, in the SZ white mica is commonly zoned, displaying partial overprint and recrystallization features (e.g. Konrad-Schmolke and Halama 2014; Regis et al. 2014; Giuntoli et al. 2018b). Halama et al. (2014) provided an improvement on this bulk approach and linked phengite ages with fluid flow and deformation events in the SZ eclogites by UV laser ^40^Ar/^39^Ar dating. One of the first applications of Lu–Hf dating of garnet investigated samples from the SZ and obtained an age of 69.2 ± 1.2 Ma (Duchêne et al. 1997). However, applicability of this method in the SZ is hampered by the polymetamorphic nature of garnet in the abundant basement rocks (e.g. Giuntoli et al. 2018b). Bulk U–Pb dating of metamorphic minerals, such as titanite, has been successfully applied to monometamorphic samples (~ 66 Ma: Ramsbotham et al. 1994; Inger et al. 1996), but this method is inadequate for dating polymetamorphic slices due to pre-Alpine inheritance (Castelli and Rubatto 2002). Despite important constraints have been obtained for the Alpine-related metamorphism with these methods, they are largely unsuitable to retrieve the pre-Alpine evolution through the investigation of polycyclic mineral relics.

In situ dating of U-Th-Pb bearing minerals has become of key importance for investigating rocks that record episodic recrystallization or partial re-equilibration stages thanks to the ability to target specific growth domains using laser ablation inductively coupled plasma mass spectrometry (LA-ICPMS) and secondary ion mass spectrometry (SIMS, or ion microprobe). Rubatto et al. (1999) reported the first in situ zircon U–Pb age data for the SZ, shading light on the Cretaceous timing of eclogite-facies metamorphism, but also constraining the ages of both protoliths and detrital components. More recent studies contributed to constrain Alpine and pre-Alpine metamorphic evolution of the SZ by in situ dating of zircon (Rubatto et al. 2011; Regis et al. 2014; Giuntoli et al. 2018a; Kunz et al. 2018) and allanite (Cenki-Tok et al. 2011; Regis et al. 2014; Giuntoli et al. 2018a). These studies have combined dating of distinct chemical domains in zoned minerals with textural and trace element analysis to link the U–Pb ages to assemblages and possibly metamorphic stages. However, the knowledge about the timing of the pre-Alpine metamorphic evolution is still limited and U-Th-Pb in situ dating constraints on zircon and allanite are only available for few localities (Fig. 1).

This study reports results obtained by combining in situ SIMS dating with LA-ICP-MS trace element composition of zircon and LA-ICP-MS allanite dating from different eclogites and metasediments from the southern and the central SZ, in order to improve our understanding of the polymetamorphic evolution of this slice of Adriatic continental crust.

## Geological setting

The SZ is the largest exposed slice of Adriatic continental crust in the Western Alps. It was part of the distal continental margin of the Adriatic plate that was separated from the European continent by the Piemonte-Liguria Ocean (e.g. Dal Piaz et al. 2001; Babist et al. 2006; Manzotti et al. 2014). It exposes a variety of metapelites, metagranitoids, mafic bodies and subordinated ultramafic bodies and marbles (e.g. Dal Piaz et al. 1972; Compagnoni 1977; Regis et al. 2015; Giuntoli and Engi 2016) (Fig. 1). Known pre-Alpine magmatic and metamorphic stages are (1) early Carboniferous gabbroic intrusions in the crystalline basement (Rubatto 1998; Rubatto et al. 1999) and associated metamorphism, which remains largely unconstrained as it was dated only in a single sample from Cima di Bonze (Regis et al. 2014), and (2) bimodal magmatism at the Carboniferous-Permian boundary, with related amphibolite to granulite-facies metamorphism (Oberhänsli et al. 1985; Lardeaux and Spalla 1991; Rubatto et al. 1999; Rebay and Spalla 2001; Giuntoli et al. 2018a). The whole sequence underwent subduction in the Late Cretaceous to Early Paleocene prior to the Alpine collision. Blueschist-facies to eclogite-facies metamorphism was followed by decompression and cooling to blueschist-facies conditions and a final greenschist-facies overprint.

The SZ has been traditionally divided into three subunits based on the different degree of HP imprint (Compagnoni 1977): (1) the Eclogitic Micaschist Complex (EMC), (2) the Gneiss Minuti Complex (GMC) and (3) the Second Dioritic-Kinzigitic Zone (IIDK) (Fig. 1). During Alpine subduction, the EMC recorded maximum P of 2.0 GPa and maximum T of 600 ± 50 °C (Compagnoni 1977; Gosso 1977; Konrad-Schmolke et al. 2006; Pognante 1989a; Tropper and Essene 1999; Zucali et al. 2002; Rubatto et al. 2011; Regis et al. 2014; Giuntoli et al. 2018b). The southern portion of the EMC was recognized to record lower peak conditions with respect to the central part (P ~ 1.6 GPa, T = 500–550 °C, Pognante, 1989a). In the EMC, phengite-schists constitute the dominant rock type, together with minor eclogites and orthogneisses. In this subunit, the eclogite mineral assemblages are well preserved, only locally overprinted by late greenschist-facies metamorphism associated to Alpine collision. Ages of HP metamorphic minerals in the EMC (Fig. 2) are ranging mainly between ~ 85 and 60 Ma (Oberhänsli et al. 1985; Ramsbotham et al. 1994; Venturini 1995; Inger et al. 1996; Duchêne et al. 1997; Ruffet et al. 1997; Rubatto et al. 1999; Dal Piaz et al. 2001; Babist et al. 2006; Cenki-Tok et al. 2011; Rubatto et al. 2011; Halama et al. 2014; Regis et al. 2014; Giuntoli et al. 2018a), with younger ages limited to Rb–Sr in mica (Ramsbotham et al. 1994; Inger et al. 1996; Ruffet et al. 1997). Most of these ages are attributed to HP conditions, and it has been proposed that this spread is due to overprinting of multiple metamorphic stages (e.g. Rubatto et al. 2011), diachronous metamorphism across slices (e.g. Regis et al. 2014; Giuntoli et al. 2018a) and recrystallization related to deformation and fluid flow affecting phengite (Halama et al. 2014). Alpine peak conditions for the GMC are 1.1–1.2 GPa and 350–500 °C (e.g. Pognante 1989a, 1989b; Giuntoli et al. 2018b). In the GMC, greenschist-facies metamorphism associated to Alpine collision pervasively overprinted the HP paragenesis and age constraints are limited (e.g. Giuntoli et al. 2018a). The IIDK consists of kilometric lenses of extensively preserved pre-Alpine amphibolites and granulites recording only local eclogite re-equilibration (e.g. Dal Piaz et al. 1971; Vuichard 1987). Further subdivisions of the SZ have been proposed after that of Compagnoni (1977), including attempts to identify different slices within the EMC (Venturini et al. 1994; Venturini 1995; Babist et al. 2006; Regis et al. 2014; Giuntoli and Engi 2016; Giuntoli 2018a, b). However, these subdivisions are mostly based on detailed studies of the central EMC only, and a common nomenclature and location of the boundaries among slices are not well established throughout the unit. Therefore, in the following we refer to the EMC as defined by Compagnoni (1977).

## Sample description

This study investigates four different areas located in the southern and central part of the EMC (Fig. 1, 2): (1) Malone Valley; (2) Ivozio Complex; (3) Chiusella Valley; and (4) Monte Mucrone. In these localities, evidence of polymetamorphism has been reported and the typical association of metasediments and eclogites is found. These features grant a diversity of pre-Alpine inheritance and a variety of rock compositions that may favour the formation of U–Pb minerals during metamorphism.

The samples collected represent the main rock types at each locality. At Monte Mucrone and Malone Valley the dominating rock types are micaschists with intercalated lenses of eclogites, whereas the Ivozio Complex is essentially made of eclogites; in the Chiusella Valley, metagabbros are overridden by pre-Alpine Mn-rich sediments (quartzites and calcschists). GPS location, full mineral assemblages and P–T conditions of each locality based on literature data are reported in Table 1. Sample locations are shown in Figs. 1 and 2; microphotos of the analysed samples are given in Additional file 1. In all the analysed samples, the dominant assemblage is composed of HP minerals, with possible pre-Alpine relics of amphibolite to granulite-facies minerals.

**Table 1:** List of the analysed samples. Mineral abbreviations are from Whitney and Evans (2010)

Sample	Location^a^	Lithology	Mineralogy^b^	Peak P–T conditions
Malone Valley				
AV16-44	N 45° 21′ 29.64″ E 09° 31′ 03.00″	Blueschist	Gln (50%) + Grt (20%) + Spn (10%) ± Ep ± Ph ± Aln ± Ap ± Cpx ± Chl (retrograde) ± Amp (retrograde) ± Zrn	~ 1.7 GPa 500 °C^c^
AV16-45	N 45° 21′ 29.64″ E 09° 31′ 03.00″	Micaschist	Qz (35%) + Ph (35%) + Grt (15%) + Pg (5%) ± Chl ± Ab ± Spn ± Aln ± Zrn	~ 1.7 GPa 500 °C^c^
AV16-51	N 45° 21′ 29.64″ E 09° 31′ 03.00″	Lws-micaschist	Qz (30%) + Wm (15%) + Chl (15%) + Czo (Lws pseudomorphs) (10%) + Grt (10%) + Gln (10%) + Spn (5%) ± Aln ± Zrn	~ 1.7 GPa 500 °C^c^
AV16-47	N 45° 21′ 29.64″ E 09° 31′ 03.00″	Eclogite	Matrix: Gln (50%) + Grt (20%) + Ep (15%) ± Ph ± Zo ± Ap ± Chl (retr.) ± Amp (retrograde) ± Ab (retrograde) ± Spn ± Zrn Boudin core: Omp (90%) ± Spn ± Amp (retr.) ± Ab (retrograde) Boudin rim: Ep (55%) + Spn (35%) ± Grt ± Zrn	~ 1.7 GPa 500 °C^c^
Ivozio Complex				
AV16-21	N 45° 32′ 09″ E 07° 50′ 46″	Eclogite	Domain 1: Grt (50%) + Omp (25%) + Gln (15%) ± Ph ± Rt ± Py Domain 2: Qz (30%) + Grt (30%) + Ph (30%) ± Ep ± Rt	1.8–2.3 GPa 550–600 °C^d^
AV16-53	N 45° 32′ 10.20″ E 07° 50′ 46.44″	Blueschist	Grt (50%) + Gln (40%) ± Cpx (relics) ± Rt ± Aln ± Ph ± Zo ± Qz ± opaques	1.8–2.3 GPa 550–600 °C^d^
AV16-57	N 45° 32′ 14.58″ E 07° 50′ 45.88″	Eclogite	Omp (50%) + Grt (25%) + Gln (15%) + Ph (15%) + Chl (retrograde) ± Rt ± Aln ± opaques	1.8–2.3 GPa 550–600 °C^d^
Chiusella Valley				
VC10-04	N 45° 33′ 03.70″ E 07° 43′ 43.20″	Mn-rich quartzite	Qz (60%) + Grt (20%) + Gln (15%) ± Aln ± Zrn	~ 1.8 GPa 550 °C^e^
Monte Mucrone				
AV17-07	N 45° 37′ 52.98″ E 07° 56′ 23.58″	Micaschist	Ph (35%) + Grt (35%) + Pg (15%) + Qz (5%) ± Rt ± Aln ± Zrn ± Chl ± Cpx (relics) ± opaques	1.3–1.5 GPa 500–600 °C^f^
AV17-16	N 45° 37′ 51.18″ E 07° 56′ 34.24″	Eclogite	Matrix: Gln (30%) + Grt (25%) + Omp (20%) + Ph (10%) + Pg (5%) ± Rt ± Ap ± Zrn Veins: Ph (40%) + Grt (30%) + Gln (20%) ± Rt ± Ep	1.3–1.5 GPa 500–600 °C^f^

^a^Coordinates refer to WGS84

^b^Modal % is based on visual estimation

^c^Pognante (1989a)

^d^Zucali et al. (2004); Zucali and Spalla (2011)

^e^Regis et al. (2014)

^f^Zucali et al. (2002) and references therein

### Malone Valley

The studied outcrop is located in the southern part of the EMC, near Alpe Mecio, ~ 2.5 km South-West of Monte Soglio. At the metre scale, strongly foliated glaucophane-epidote-rich schists are associated with garnet-bearing micaschists and quartz layers (Vho et al. 2020) in which lozenge-shape aggregates of zoisite are preserved and are interpreted as lawsonite pseudomorphs, as already proposed by Pognante (1989a). Decimetric eclogite boudins are found within the schists and are typically surrounded by an epidote-rich, cm-thick rim. Two metasediment and one eclogite samples collected at this outcrop are investigated.

Sample AV16-44 is a fine-grained glaucophane-epidote-schist. It contains two types of garnet: (1) sub-millimetric garnet grains that are rich in epidote inclusions and are distributed within the foliation and (2) plurimillimeter garnet porphyroblasts surrounded by the foliation, preserving a porphyroclastic fractured core and a rim rich in epidote inclusions. Sample AV16-45 is a quartz-rich, garnet-bearing micaschist with phengite, paragonite, chlorite and allanite defining a pervasive foliation that wraps around mm-sized garnets. Sample AV16-51 is a micaschist with a strong foliation marked by phengite and chlorite that wraps plurimillimetric garnet grains and lozenge-shaped aggregates of clinozoisite and quartz interpreted as pseudomorphs after lawsonite. Sample AV16-47 is a mafic boudin embedded in the metasediments. The matrix is weakly foliated, dominated by glaucophane, epidote and euhedral garnet crystals with a size of 100–500 μm. It contains lenses of omphacite surrounded by epidote.

### Ivozio Complex

The Ivozio Complex is located at the southeastern edge of the SZ, near the village of Ivozio. It consists of a metagabbro body with a diameter of ~ 500 m, associated to scarce ultramafic rocks and surrounded by micaschists (Pognante et al. 1980; Zucali et al. 2004; Delleani et al. 2018). Mafic lithologies have fully recrystallized during Alpine HP metamorphism and mainly consist of glaucophane, omphacite and garnet in various proportions, with subordinate phengite, zoisite and quartz; lozenge-shape aggregates of zoisite and paragonite, which are interpreted as lawsonite pseudomorphs, are locally present (Zucali et al. 2004; Zucali and Spalla 2011). In the studied samples, zircon cores are the only pre-Alpine mineral relics.

Eclogite sample AV16-21 consists of two domains: (1) a glaucophane-bearing eclogite, dominated by pale rose, inclusion-poor garnet and omphacite, with minor rutile, phengite and pyrite, and (2) a quartz-rich domain containing mm-size phengite crystals and garnet; the garnet has an inclusion-rich core (mainly quartz, phengite and rutile) and an inclusion-free rim. Sample AV16-53 is a glaucophane-garnet-fels with garnet grains from 1 mm up to 2 cm in size. Glaucophane grains are euhedral and have sharp and well-preserved contacts with garnet. Relics of clinopyroxene are locally preserved. Eclogite AV16-57 contains mainly omphacite and garnet; locally phengite veins and enrichment in glaucophane and phengite occurs. In these domains, glaucophane grains are pale blue in colour, with an irregular shape and retrogression to green amphibole along the rims, garnet is strongly fractured with locally resorbed rims and omphacite is present as relic phase.

### Chiusella Valley

In the Chiusella Valley, located West of Cima di Bonze, coarse grained metagabbro is overridden by a few meters of Mn-rich quartzite and micaschist locally interbedded with metabasalt and metagabbro. Sample VC10-04 was collected near Alpe Solanger. It is a Mn-rich quartzite, characterized by a pervasive millimetre to submillimetric foliation that is marked by iso-oriented elongated glaucophane blasts and quartz and garnet layers. It contains pink garnet crystals with atoll texture and accessory allanite and zircon.

### Monte Mucrone

Monte Mucrone is the northernmost area investigated and is dominated by micaschists and paragneisses that include centimetric to metric mafic lenses and boudins. The metasediments locally preserve relics of migmatitic textures. Samples were collected between the Albergo Savoia and Bocchetta del Lago.

Sample AV17-07 is a micaschist containing quartz, several hundreds of microns size phengite and lozenge-shape aggregates of fine-grained paragonite, whose shape resembles that of lawsonite pseudomorphs. Garnet occurs as millimetre porphyroclastic cores surrounded by euhedral, smaller garnets and as submillimetre euhedral grains. Locally garnet has atoll textures with quartz and coarse grained phengite filling the central part. Sample AV17-16 is an eclogite composed of glaucophane, garnet, omphacite, paragonite, phengite and minor rutile, which contains veins and pods of oriented coarse grained phengite, garnet and glaucophane.

## Analytical methods

### Mineral separation and grain mount preparation

Samples were disaggregated using a SELFRAG apparatus (Institute of Geological Sciences, University of Bern), which produces a high yield of intact mineral grains by high-voltage pulsing, and sieved to select the grain fraction between 64 and 250 μm. Zircon and allanite grains were separated using conventional magnetic and density-based techniques, hand-picked, mounted in epoxy resin or acrylic and manually polished down to expose the near equatorial section.

### SEM imaging

Back scattered electron (BSE) images of allanite were obtained from polished thin section with a ZEISS EVO 50 scanning electron microscope at the Institute of Geological Sciences (University of Bern) using a voltage of 20 kV, current of ~ 1 nA and a working distance of 10 mm. Zircon charge contrast (CC) and BSE images of allanite in grain mount were taken with the same instrument at 10–16 kV accelerating voltage, variable pressure (VP) conditions and 10 mm working distance. It has been demonstrated that CC images correlate exactly to cathodoluminescence images and result from the complex interaction between the electron beam, the positive ions generated by electron‐gas interactions in the chamber, a biased detector, and the sample (Griffin 2000; Watt et al. 2000). Internal check in the Bern laboratory confirmed that CC images are identical to panchromatic cathodoluminescence images, but have the advantage to require no coating of the sample.

### LA-ICP-MS trace element analysis

Trace element analyses of zircon were performed at the Institute of Geological Sciences (University of Bern) using two different instruments: (1) a LA-ICP-MS Geolas Pro 193 nm ArF excimer laser coupled to an Elan DRC-e quadrupole ICP-MS (samples AV16-21, AV16-44, AV16-47) and (2) a RESOlution Laser System coupled to an Agilent 7900 quadrupole ICP-MS (samples AV16-45, AV16-51, AV17-07, AV17-16). A He-H_2_ gas mixture was used as the aerosol transport gas. Allanite and zircon trace element analyses were performed with laser beam diameters of 20 and 24 μm, frequencies of 9 and 5 Hz and energy densities on the sample of 2.5 and 4.0 J•cm^−2^. Sample analyses were calibrated using NIST SRM 610 and 612 (Jochum et al. 2011) and accuracy was monitored using the reference material BCR-2 g and GSD-1Gg (Jochum et al. 2005). Data reduction was performed using the SILLS software package (Guillong et al. 2008; samples AV16-21, AV16-44, AV16-47) and the software Iolite (Hellstrom et al. 2008; Paton et al. 2011; samples AV16-45, AV16-51, AV17-07, AV17-16). Further information on the instrument setup is reported in Additional file 2 (Tables AF2-T1, AF2-T2).

### Allanite LA-ICP-MS dating

Allanite was dated on polished thin sections and on separate single grains mounted in acrylic. In situ analyses were performed using a LA-ICP-MS Geolas Pro 193 nm ArF excimer laser coupled to an Elan DRC-e quadrupole ICP-MS at the Institute of Geological Sciences (University of Bern). The analytical procedure is described in detail in Burn et al. (2017). Spot sizes were chosen at 24 μm. Frequency used was 9 Hz and energy density on the sample 2.5 J•cm^−2^. NIST SRM 610 (Jochum et al. 2011) measurements were performed for quantification of U- and Th-concentrations. Plešovice zircon (337.13 ± 0.37 Ma, Sláma et al. 2008) was used as primary standard. The quality of the data was monitored using secondary allanite standards (CAP: 275 ± 1.5 Ma, Barth et al. 1994; SISS 31.5 ± 0. 5 Ma, von Blackenburg 1992). Data reduction was performed with the in-house program TRINITY (Burn et al. 2017). Common lead correction on single analyses was performed based on ^207^Pb according to the procedure of Gregory et al. (2007) as updated in Burn et al. (2017). Th-Pb ages obtained for CAP secondary standards for each measurement session overlap with the reference value within uncertainty (calculated ages between 277 ± 5 and 282 ± 7 Ma); SISS Th-Pb ages overlap with the reference value for each session (calculated ages between 31.1 ± 1.0, 29.0 ± 1.7 Ma and 29.9 ± 0.7 Ma) (for details see Additional file 2, Table AD2-T3). Given the relatively high percept of initial Pb in the allanite analyses (typically 40–100%), the more robust age for a statistically consistent data population is obtained in most cases with a regression in the Tera-Wasserburg (TW) diagram using uncorrected ratios, thus avoiding having to assume an initial Pb composition. Therefore, the reported values are obtained following this strategy when not differently specified. Results are given at 95% confidence limit. Within individual samples, the Th-Pb dates were concordant with the U–Pb dates, but were more dispersed.

### Zircon ion microprobe and LA-ICP-MS dating

Zircon grains in all samples except for VC10-04 were analysed for U, Th and Pb using the Cameca IMS 1280HR ion microprobe instrument at the SwissSIMS facility (University of Lausanne, Switzerland). An O^2−^ primary beam was used with a 3–7 nA current and focused to a spot size of 20–25 μm. Secondary ions were extracted at 10 kV, a mass resolution power M/ΔM ~ 5000 at 10% of the peak height, and an energy window of 50 eV. Run table and analytical conditions were similar to those previously described for zircon U-Th-Pb analysis by Whitehouse and Kamber (2005). Reference zircon Temora (416.75 ± 0.24 Ma, Black et al. 2003) was used as primary standard during calibration, using a UO_2_/U vs. Pb/U relative sensitivity calibration; the calibration uncertainty was between 1.15 and 1.87% for each analytical session. Reference zircon Plešovice (337.13 ± 0.37 Ma, Sláma et al. 2008) was used as secondary standard and returned Concordia ages within 1% of the reference value in each analytical session (335.2 ± 8.3 Ma and 338.0 ± 3.2 Ma). Data reduction was carried out using the CAMECA-CIPS software compiled by Martin Whitehouse (analytical session 1, samples AV16-21, AV16-44, AV16-47) and SQUID 2.50 (add-in for Microsoft Excel; Ludwig 2009) (analytical session 2, samples AV16-45, AV16-51, AV17-07 and AV17-16). Common Pb correction was based on the measured ^204^Pb signal (when significant relative to background) assuming the present day model terrestrial Pb composition of Stacey and Kramers (1975). A ^208^Pb-based correction (Williams 1998) was applied to ages younger than 100 Ma low in Th/U zircon (sample AV17-07). Calculations of Concordia and weighted ages and plots were done using Isoplot 4.15 (add-in for Microsoft Excel; Ludwig 2003). Age calculations use the decay constant recommendations of Steiger and Jäger (1977). To account for external errors, uncertainties on average ages were forced to be at least 1%. Average ages are given at 95% confidence limit if not otherwise specified.

U–Pb analyses of zircon of sample VC10-04 were obtained by LA-ICP-MS at the Institute of Geological Sciences (University of Bern). The analyses were performed using a LA-ICP-MS Geolas Pro 193 nm ArF excimer laser coupled to an Elan DRC-e quadrupole ICP-MS following the strategy described in Manzotti et al. (2012). The ablation was performed with an energy density on the sample of 5 J•cm^−2^ using beam diameters of 25 μm. A He-H_2_ gas mixture was used as the aerosol transport gas. Zircon reference material GJ-1 (608.53 ± 0.37 Ma, Jackson et al. 2004) was used to correct for elemental fractionation and instrumental mass bias. NIST SRM 610 (Jochum et al. 2011) was used for the quantification of concentrations, using ^29^Si as an internal standard. Secondary standards Plešovice zircon (337.13 ± 0.37 Ma, Sláma et al. 2008) and 91,500 zircon (1065 Ma, Wiedenbeck et al. 1995) returned ages within 1% of the reference value. Data processing was performed offline using the Lamtool version 081117 following the procedure detailed in Manzotti et al. (2012). Calculations of Concordia and weighted ages and plots were done using Isoplot 4.15 (add-in for Microsoft Excel; Ludwig 2003). To account for external errors, uncertainties on average ages were forced to be at least 1%. Average ages are given at 95% confidence limit if not otherwise specified.

## Results

### Malone Valley

#### Blueschist AV16-44

**Fig. 3:**
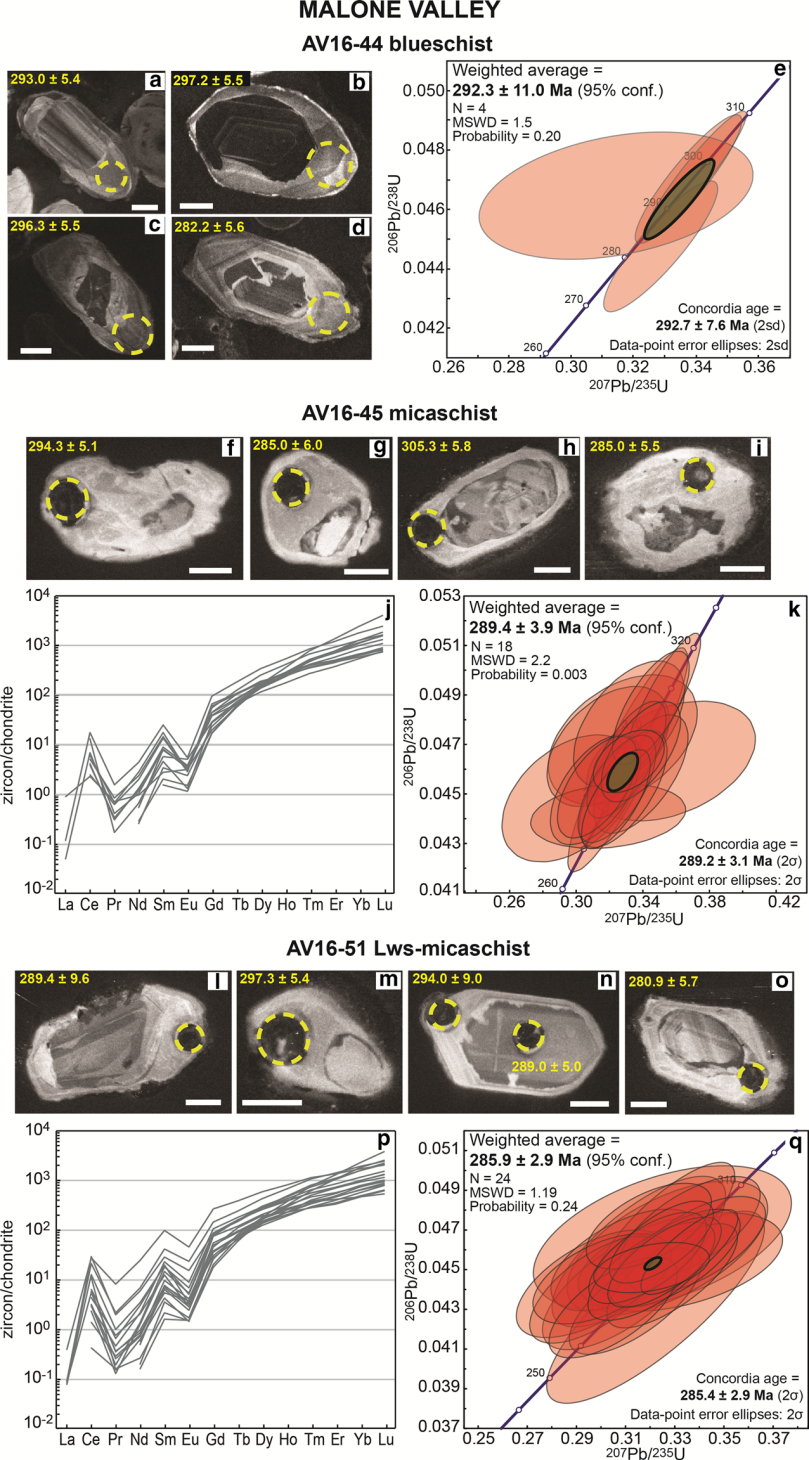
Zircon in metasediments from Malone Valley. Blueschist AV16-44: **a**–**d** CC images of the analysed zircon grains. Scale bar in all images is 30 μm. Measured spots are shown (yellow circles) with the associated date (± 1σ Ma). **e** Concordia plot of zircon rim dates and weighted average ^206^Pb/^238^U age. The green circle represents the calculated Concordia age. Micaschist AV16-45: **f**–**i** Selected CC images of analysed zircon grains. Scale bar in all images is 30 μm. Measured spots are shown (yellow circles) with the associated date (± 1σ Ma). **j** REE patterns of zircon rims. **k** Concordia plot of zircon rim dates and weighted average ^206^Pb/^238^U age. The green circle represents the calculated Concordia age. Lws-micaschist AV16-51: **l**–**o** Selected CC images of analysed zircon grains. Scale bar in all images is 30 μm. Measured spots are shown (yellow circles) with the associated date (± 1σ, Ma). **p** REE patterns of zircon rims. **q** Concordia plot of zircon rim dates and weighted average ^206^Pb/^238^U age. The green circle represents the calculated Concordia age

Zircon grains have a variable shape and are characterized by the presence of rounded cores with various zoning patterns occasionally overgrown by a grey, weakly zoned rim of maximum width of ~ 30 μm, but typically < 10 μm thick (Fig. 3). Two core analyses result in ^206^Pb/^238^U dates of 645 ± 12 Ma and 604 ± 11 Ma; along with the internal crystal morphology, this suggests a detrital origin (Table 2). Due to the small size and rare occurrence of rims, only four spots could be measured and return a weighted average age of 292 ± 11 Ma (Fig. 3, Additional file 3). The Th/U value for the rim is 0.03–0.13.

**Table 2:** U, Th and Pb SIMS zircon data of the eclogite AV16-21 from Ivozio Complex, the eclogite AV16-47 and the blueschist AV16-44 from Malone Valley

Spot name	U (µg/g)	Th (µg/g)	Th/U	f206^a^	Concordia diagram (^204^Pb corr.)					^206^Pb/^238^U Age (Ma)	± 1σ	Comments
					^207^Pb/^235^U	± 1σ%	^206^Pb/^238^U	± 1σ%	ρ			
Ivozio Complex–AV16-21 eclogite												
@12	2797	190	0.07	0.13	0.3787	1.95	0.05159	1.87	0.9616	324.3	5.9	Core
@15	1224	59	0.05	0.02	0.3859	1.98	0.05267	1.91	0.9619	330.9	6.2	Core
@7	4281	793	0.19	0.10	0.3973	1.98	0.05352	1.87	0.9449	336.1	6.1	Core
@14	3151	228	0.07	0.01	0.3958	1.90	0.05383	1.88	0.9888	338.0	6.2	Core
@6	495	15	0.03	0.78	0.4035	5.99	0.05449	1.87	0.3128	342.0	6.2	Core
@5	871	94	0.11	0.39	0.3957	3.41	0.05453	1.87	0.5499	342.3	6.2	Core
@16	3467	257	0.07	0.01	0.4004	1.91	0.05470	1.89	0.9898	343.3	6.3	Core
@8	2198	146	0.07	0.17	0.4017	2.29	0.05523	1.90	0.8279	346.6	6.4	Core
@13	2000	123	0.06	0.02	0.4139	1.89	0.05635	1.87	0.9921	353.4	6.4	Core
@1	2910	208	0.07	0.02	0.4203	1.89	0.05660	1.87	0.9888	354.9	6.5	Core
* @10*	*4297*	*334*	*0.08*	*0.02*	*0.4389*	*1.89*	*0.05964*	*1.88*	*0.9964*	*373.4*	*6.8*	*Possible inheritance*
* @11*	*1096*	*87*	*0.08*	*0.02*	*0.4570*	*2.16*	*0.06246*	*2.04*	*0.9473*	*390.5*	*7.7*	*Possible inheritance*
* @2*	*95*	*43*	*0.45*	*0.23*	*0.5007*	*2.74*	*0.06593*	*1.89*	*0.6887*	*411.6*	*7.5*	*Possible inheritance*
Malone Valley–AV16-44 blueschist												
@6	357	24	0.07	0.10	0.3286	2.28	0.04475	2.02	0.8849	282.2	5.6	Rim
@1	41	1	0.03	1.23	0.3143	5.73	0.04650	1.88	0.3284	293.0	5.4	Rim
@7	311	20	0.06	0.16	0.3376	2.11	0.04703	1.90	0.9042	296.3	5.5	Rim
@5	496	64	0.13	0.07	0.3399	2.01	0.04718	1.88	0.9389	297.2	5.5	Rim
* @3*	*77*	*30*	*0.39*	*0.13*	*0.8102*	*2.19*	*0.09825*	*1.91*	*0.8714*	*604.1*	*11.0*	*Core*
* @2*	*49*	*27*	*0.56*	*0.52*	*0.9077*	*2.97*	*0.10530*	*1.88*	*0.6322*	*645.4*	*11.6*	*Core*
Malone Valley–AV16-47 eclogite												
* @11*	*9*	*2*	*0.21*	*4.49*	*0.2492*	*27.62*	*0.03858*	*2.53*	*0.0916*	*244.0*	*6.1*	*Possible Pb loss*
@16	8	3	0.35	3.29	0.2370	38.35	0.04164	1.95	0.0507	263.0	5.0	
@8	6	1	0.21	3.07	0.2882	25.48	0.04221	2.09	0.0819	266.5	5.4	
@13	9	3	0.36	2.55	0.2792	23.51	0.04331	2.00	0.0850	273.3	5.4	
@15	6	3	0.45	3.32	0.3055	19.83	0.04344	1.98	0.0998	274.1	5.3	
@14	7	2	0.32	2.35	0.2919	18.63	0.04351	2.13	0.1141	274.5	5.7	
@18	16	3	0.20	2.41	0.2493	20.12	0.04465	2.02	0.1002	281.6	5.6	
@7	16	5	0.28	1.52	0.3323	13.06	0.04484	1.94	0.1483	282.8	5.4	
@2	15	2	0.16	0.62	0.3214	8.33	0.04489	1.93	0.2322	283.1	5.4	
@6	11	5	0.41	1.63	0.3190	12.89	0.04497	1.91	0.1481	283.6	5.3	
@4	16	6	0.36	1.71	0.3125	18.19	0.04510	1.95	0.1072	284.4	5.4	
@17	323	34	0.11	0.08	0.3241	2.04	0.04540	1.88	0.9252	286.2	5.3	
@3	10	1	0.15	2.04	0.3043	20.62	0.04547	1.93	0.0936	286.7	5.4	
@19	6	1	0.20	3.26	0.3110	31.62	0.04550	1.88	0.0594	286.9	5.3	
@12	9	4	0.42	1.57	0.3485	14.47	0.04553	1.89	0.1304	287.0	5.3	
@20	14	6	0.44	2.12	0.3099	16.01	0.04574	1.89	0.1181	288.3	5.3	
@10	8	2	0.27	3.35	0.3028	22.59	0.04577	1.92	0.0848	288.5	5.4	
@1	7	3	0.38	2.74	0.2845	27.23	0.04581	1.91	0.0701	288.8	5.4	
@9	7	2	0.30	1.69	0.3783	16.51	0.04608	1.93	0.1167	290.4	5.5	
@5	10	2	0.18	2.49	0.3107	24.39	0.04657	2.13	0.0871	293.4	6.1	
Ivozio Complex–AV16-21 eclogite												
@12	2797	190	0.07	0.13	0.3787	1.95	0.05159	1.87	0.9616	324.3	5.9	Core
@15	1224	59	0.05	0.02	0.3859	1.98	0.05267	1.91	0.9619	330.9	6.2	Core
@7	4281	793	0.19	0.10	0.3973	1.98	0.05352	1.87	0.9449	336.1	6.1	Core
@14	3151	228	0.07	0.01	0.3958	1.90	0.05383	1.88	0.9888	338.0	6.2	Core
@6	495	15	0.03	0.78	0.4035	5.99	0.05449	1.87	0.3128	342.0	6.2	Core
@5	871	94	0.11	0.39	0.3957	3.41	0.05453	1.87	0.5499	342.3	6.2	Core
@16	3467	257	0.07	0.01	0.4004	1.91	0.05470	1.89	0.9898	343.3	6.3	Core
@8	2198	146	0.07	0.17	0.4017	2.29	0.05523	1.90	0.8279	346.6	6.4	Core
@13	2000	123	0.06	0.02	0.4139	1.89	0.05635	1.87	0.9921	353.4	6.4	Core
@1	2910	208	0.07	0.02	0.4203	1.89	0.05660	1.87	0.9888	354.9	6.5	Core
* @10*	*4297*	*334*	*0.08*	*0.02*	*0.4389*	*1.89*	*0.05964*	*1.88*	*0.9964*	*373.4*	*6.8*	*Possible inheritance*
* @11*	*1096*	*87*	*0.08*	*0.02*	*0.4570*	*2.16*	*0.06246*	*2.04*	*0.9473*	*390.5*	*7.7*	*Possible inheritance*
* @2*	*95*	*43*	*0.45*	*0.23*	*0.5007*	*2.74*	*0.06593*	*1.89*	*0.6887*	*411.6*	*7.5*	*Possible inheritance*

**Fig. 4:**
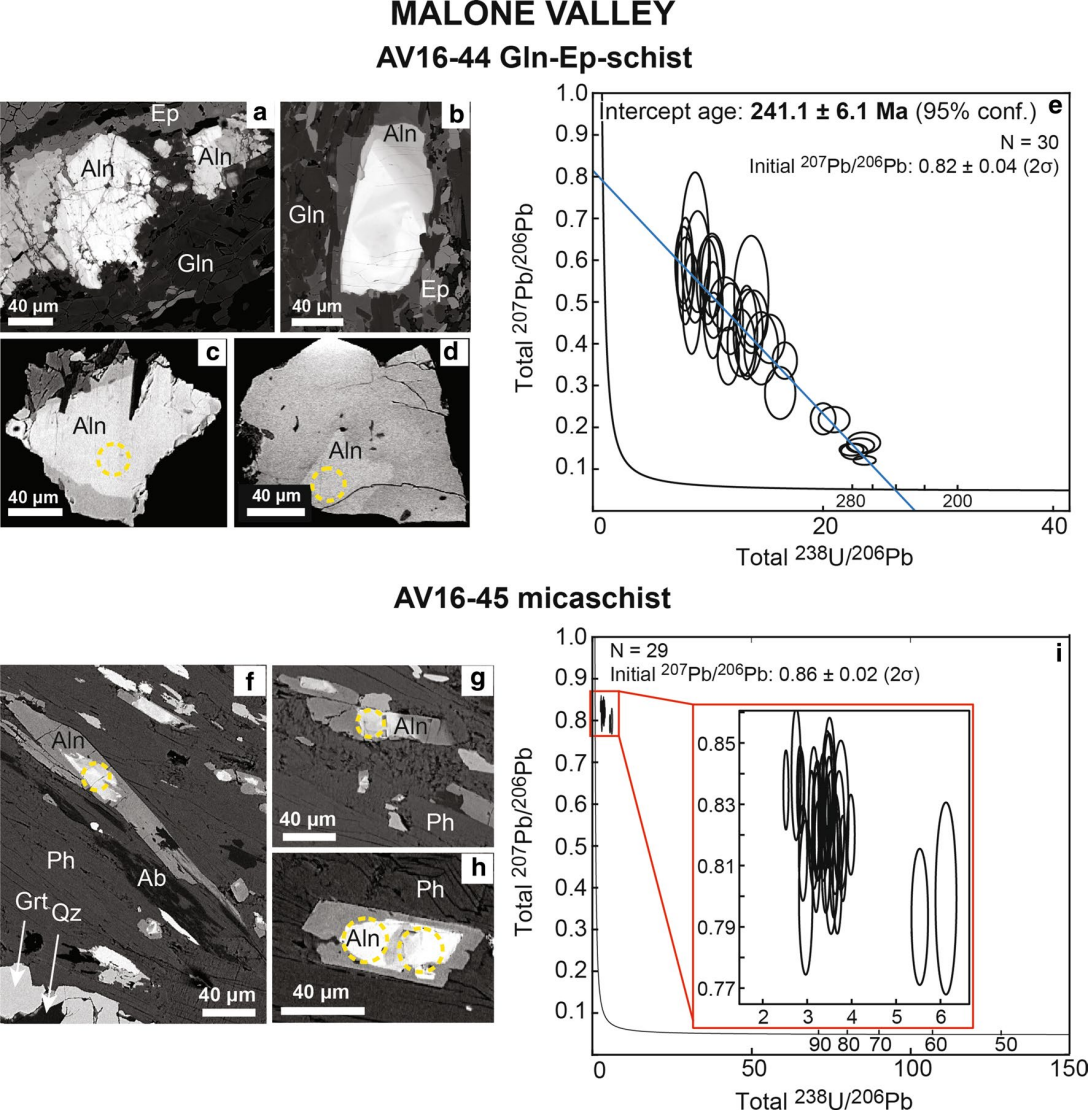
Allanite in samples from Malone Valley. Blueschist AV16-44: **a**, **b** Thin section BSE images of allanite (Aln) associated to glaucophane (Gln) and epidote (Ep). **c**, **d** BSE images of allanite (Aln) in a grain mount. **e** Tera-Wasserburg plot for LA-ICP-MS U–Pb allanite analyses. Data-point error ellipses are 2σ. Micaschist AV16-45: **f**–**h** Thin section BSE images of allanite (Aln) associated to phengite (Ph) in the main foliation. Garnet (Grt) and quartz (Qz) are part of the HP mineral assemblage; albite (Ab) is retrograde. **i** Tera-Wasserburg plot for LA-ICP-MS U–Pb allanite analyses. Data-point error ellipses are 2σ

Data were processed using the CAMECA-CIPS software. The dates that were not used for the age calculations shown in Figs. 3, 4 and 7 are reported in italics

^a^$$\mathrm{f}206= \frac{{}^{206}{\mathrm{Pb}}_{\mathrm{common}}}{{}^{206}{\mathrm{Pb}}_{\mathrm{total}}}*100$$

**Fig. 5:**
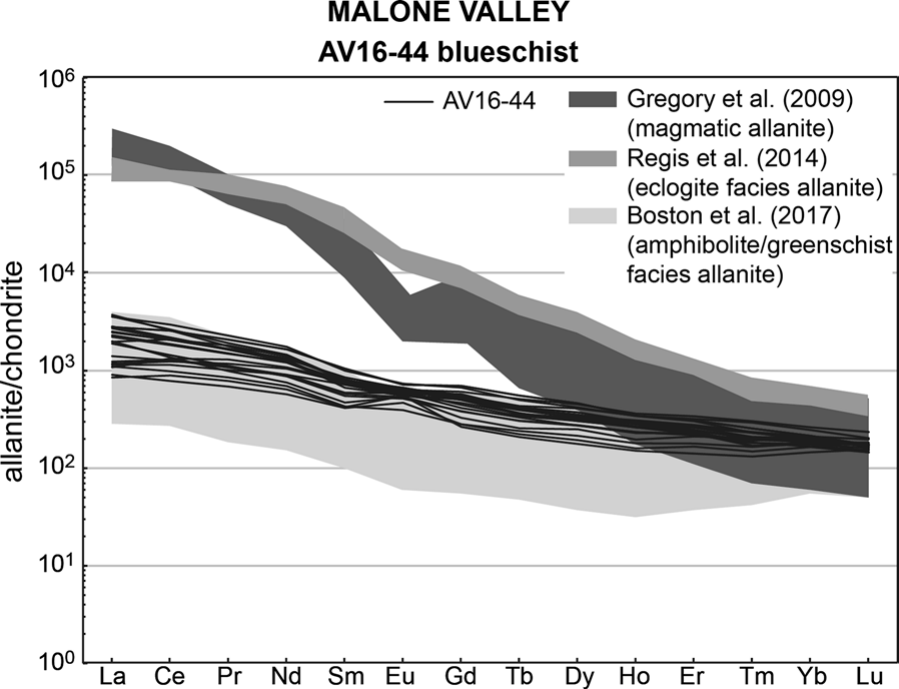
Allanite REE patterns in Blueschist AV16-44 from Malone Valley (black lines). REE patterns are compared to those of magmatic allanites from the Bergell Pluton (Gregory et al. 2009), of HP allanites in a metapelite from the Mombarone area of the EMC (Regis et al. 2014) and of amphibolite to greenschist facies allanite rims in a calcschist from Campolungo in the Central Alps (Boston et al. 2017)

Allanite was found in the matrix as grains with a dimension ranging from ~ 60 to ~ 250 μm, characterized by a core brighter in BSE images and a darker rim (Fig. 4). The grains are usually fractured, making impossible to separate whole allanite grains from the sample. Th/U ratio ranges between 0.4 and 14.3 (Table 3). On the TW diagram, the allanite data spread along a single mixing line (initial ^207^Pb/^206^Pb = 0.82 ± 0.04) that intercepts the Concordia at 241.1 ± 6.1 Ma. The REE patterns of this allanite are relatively flat with a minor enrichment in LREE (La_N_/Lu_N_ = 4.9–21.2) and no significant Eu anomaly (Fig. 5, Additional file 4).

**Table 3:** U, Th and Pb LA-ICP-MS allanite data for the Malone Valley samples

Spot name	Th/U	f206^a^	f208^b^	^238^U/^206^Pb total	± 1σ%	^208^Pb/^232^Th total	± 1σ%	^207^Pb/^206^Pb total	± 1σ%	^206^Pb/^208^Pb total	± 1σ%
AV16-44 blueschist											
a10	3.3	0.61	0.92	13.7648	5.45	0.06875	5.45	0.5180	13.56	0.7234	9.72
a11	1.8	0.15	0.61	23.5382	3.06	0.02611	3.58	0.1631	7.65	1.9886	6.86
a12	3.1	0.49	0.88	13.4950	4.72	0.05999	3.77	0.4261	12.16	0.8613	4.94
a13	14.3	0.44	0.48	14.6266	5.06	0.02084	3.03	0.3892	11.16	0.5164	5.02
a15	1.0	0.13	0.73	22.4747	2.33	0.03271	3.63	0.1465	4.90	2.7610	4.33
a16	14.3	0.66	0.66	10.3774	4.61	0.02817	2.45	0.5603	8.66	0.4746	3.48
b7	3.3	0.49	0.84	13.3672	3.47	0.05465	3.33	0.4269	13.13	0.8181	4.57
b8	10.0	0.68	0.80	8.0391	4.36	0.04410	3.01	0.5741	11.12	0.5616	4.43
b9	12.5	0.56	0.61	10.4430	4.08	0.02780	3.05	0.4807	12.00	0.5226	4.25
b10	2.8	0.71	0.97	9.1026	5.95	0.12270	5.30	0.5967	10.13	0.6520	7.02
b11	4.2	0.75	0.96	8.8859	6.75	0.09793	4.47	0.6303	14.27	0.6063	7.85
b12	4.8	0.53	0.80	11.9325	4.70	0.05963	4.46	0.4598	9.23	0.7156	6.12
b13	1.3	0.14	0.68	23.2523	2.58	0.02971	3.40	0.1564	5.54	2.3456	4.46
b15	1.1	0.09	0.57	23.6168	2.07	0.02476	2.99	0.1209	4.39	2.9689	3.47
b16	5.9	0.22	0.40	20.9605	3.24	0.01846	3.16	0.2183	6.97	0.8871	4.17
b17	1.2	0.30	0.82	16.2748	4.08	0.08076	4.04	0.2821	11.05	1.3023	5.66
c7	2.7	0.40	0.81	16.6723	3.21	0.04666	2.82	0.3597	6.08	0.9675	3.66
c9	6.7	0.42	0.63	11.7679	3.96	0.03527	2.79	0.3708	9.23	0.7208	4.38
c11	2.7	0.65	1.02	9.9727	4.78	0.09992	3.23	0.5487	9.30	0.7478	6.33
c13	7.7	0.46	0.62	12.8828	4.63	0.03026	2.77	0.4021	9.97	0.6494	4.78
c14	4.8	0.58	0.82	8.5835	4.57	0.07413	3.96	0.4954	12.08	0.6754	8.67
c15	6.7	0.53	0.77	13.9947	4.33	0.03257	2.98	0.4599	7.35	0.6894	6.13
c16	0.4	0.12	0.83	22.7153	2.54	0.05996	4.07	0.1421	6.66	3.3315	4.59
d7	3.1	0.69	1.01	10.3506	5.23	0.08724	2.78	0.5820	7.02	0.6947	4.78
d8	12.5	0.54	0.58	10.4834	3.66	0.02749	1.72	0.4680	5.79	0.5080	3.52
d9	9.1	0.22	0.31	20.0085	2.71	0.01661	1.80	0.2203	8.48	0.6622	2.95
d11	3.6	0.70	0.92	7.8944	4.64	0.10729	3.47	0.5882	7.79	0.6271	5.50
d14	11.1	0.67	0.70	8.0436	5.03	0.04244	2.77	0.5668	7.74	0.5025	5.58
d15	11.1	0.47	0.52	15.3645	4.28	0.02046	2.46	0.4117	7.06	0.5247	4.49
d16	4.8	0.58	0.86	11.4948	6.01	0.05057	3.91	0.4956	6.42	0.7121	8.18
AV16-45 micaschist											
d7	7.7	0.99	0.98	2.8709	1.13	0.16335	2.69	0.8345	0.77	0.4772	0.78
d8	14.3	0.98	0.95	3.4529	1.12	0.07380	2.64	0.8248	0.90	0.4683	0.81
d9	12.5	0.97	0.96	3.2476	1.22	0.08446	2.66	0.8184	0.77	0.4762	0.79
d10	6.3	0.96	0.97	3.6785	1.37	0.14824	2.82	0.8074	1.22	0.4859	1.19
d11	9.1	0.98	0.97	3.2270	1.45	0.12051	2.59	0.8265	1.08	0.4743	1.16
d12	8.3	0.95	0.98	2.9663	2.57	0.13550	2.87	0.7996	1.58	0.4981	1.89
d13	10.0	0.98	0.96	3.7171	1.57	0.09245	2.85	0.8233	1.06	0.4743	1.08
d14	16.7	0.98	0.96	3.0976	1.18	0.07036	2.58	0.8240	1.03	0.4708	0.90
d15	12.5	0.99	0.97	3.4491	1.69	0.08128	2.67	0.8333	1.24	0.4701	1.36
d16	12.5	0.98	0.97	3.2288	1.48	0.09037	2.89	0.8227	1.07	0.4759	1.01
e7	10.0	0.97	0.97	3.2008	1.47	0.10187	2.83	0.8187	1.46	0.4795	1.20
e8	12.5	1.00	0.97	3.4901	1.71	0.07706	3.08	0.8359	1.03	0.4687	0.93
e10	10.0	0.98	0.97	3.3608	1.67	0.10205	3.29	0.8258	1.26	0.4763	1.07
e12	11.1	0.98	0.97	3.9826	1.09	0.07643	2.72	0.8201	0.80	0.4784	0.82
e13	7.7	0.97	0.96	4.2551	1.79	0.11238	2.74	0.8156	1.44	0.4773	1.33
e14	14.3	0.98	0.97	3.1493	1.88	0.07585	2.88	0.8255	1.40	0.4764	1.37
a10	11.1	0.96	0.95	2.9047	1.61	0.11399	3.00	0.8141	0.96	0.4754	0.96
a11	6.3	0.95	0.98	6.1239	1.93	0.08546	3.28	0.7993	1.96	0.4962	1.05
a12	12.5	0.98	0.97	3.7571	2.31	0.07317	3.01	0.8264	1.48	0.4735	1.42
a13	9.1	0.99	0.95	3.5025	1.97	0.11581	3.16	0.8337	1.47	0.4641	1.52
a14	20.0	0.98	0.93	3.4058	1.31	0.05169	2.85	0.8226	0.87	0.4579	0.76
a15	5.6	0.97	0.98	7.7891	4.30	0.07928	6.16	0.8148	2.29	0.4882	2.73
b7	10.0	0.96	0.93	3.5370	2.11	0.09671	5.42	0.8143	1.63	0.4658	1.63
b8	11.1	0.96	0.95	3.6024	1.35	0.07926	4.59	0.8117	1.15	0.4725	0.92
b12	11.1	0.99	0.96	2.8421	1.29	0.11103	4.54	0.8348	0.85	0.4669	0.86
b13	16.7	0.96	0.95	3.4192	1.28	0.05859	4.54	0.8088	0.88	0.4740	0.92
b14	11.1	0.94	0.94	5.5336	1.69	0.05528	4.63	0.7933	1.40	0.4826	1.50
b15	14.3	0.97	0.96	3.6286	1.54	0.06453	4.57	0.8171	1.00	0.4742	1.01
b16	16.7	0.99	0.96	2.5220	1.17	0.08487	4.52	0.8347	0.77	0.4653	0.82

^a^$$\mathrm{f}206= \frac{{}^{206}{\mathrm{Pb}}_{\mathrm{common}}}{{}^{206}{\mathrm{Pb}}_{\mathrm{total}}}$$

^b^$$\mathrm{f}208= \frac{{}^{208}{\mathrm{Pb}}_{\mathrm{common}}}{{}^{208}{\mathrm{Pb}}_{\mathrm{total}}}$$

#### Micaschist AV16-45

Zircon grains are composed of a core with variable zoning that is overgrown by a light grey, unzoned rim of maximum width of ~ 20 μm (Fig. 3). Zircon rims have low Th/U, between 0.01 and 0.05 (Table 4). Eighteen analyses on zircon rims yield an early Permian age of 289.4 ± 3.9 Ma. The REE patterns of the rims show enrichment in HREE (Gd_N_/Lu_N_ = 0.01–0.08, Fig. 3 and Additional file 4) and are characterized by a variable Eu negative anomaly (Eu/Eu* = 0.07–0.30). Zircon rim contains 3.4–20 µg/g Ti that results in Ti-in-zircon temperature values scattering between ~ 655 and ~ 810 °C (Additional file 5). However, titanite is the Ti-bearing phase in the HP assemblage, and in absence of rutile relics, these have to be interpreted as minimum temperatures.

**Table 4:** U, Th and Pb SIMS zircon data of the micaschist AV16-45, the Lws-micaschist 16–51 and the eclogite AV16-47 from Malone Valley, and the micaschist AV17-07 and the eclogite AV17-16 from Monte Mucrone

Spot name	U (µg/g)	Th (µg/g)	Th/U	f206^a^	Concordia diagram (^204^Pb corr.)					^206^Pb/^238^U Age (Ma)	± 1σ	Comments
					^207^Pb/^235^U	± 1σ%	^206^Pb/^238^U	± 1σ%	ρ			
Malone Valley–AV16-45 micaschist												
* @16*	*49*	*1*	*0.02*	*1.43*	*0.2968*	*6.29*	*0.03992*	*2.00*	*0.3187*	*252.3*	*5.0*	*Possible Pb loss*
@9	314	4	0.01	2.02	0.3194	5.96	0.04411	1.21	0.2031	278.3	3.3	Rim
@12	417	2	0.00	0.26	0.3241	2.61	0.04479	1.82	0.6997	282.5	5.0	Rim
@11	209	4	0.02	1.22	0.2959	5.57	0.04482	2.34	0.4189	282.7	6.5	Rim
@20	253	3	0.01	0.50	0.3118	3.57	0.04517	2.01	0.5622	284.8	5.6	Rim
@18	283	4	0.01	0.27	0.3246	2.95	0.04521	1.96	0.6637	285.0	5.5	Rim
@8	240	3	0.01	0.32	0.3283	3.29	0.04521	2.13	0.6481	285.0	6.0	Rim
@14	344	5	0.01	0.46	0.3128	3.12	0.04528	1.85	0.5938	285.5	5.2	Rim
@10	236	3	0.01	0.39	0.3390	3.41	0.04580	2.13	0.6250	288.7	6.0	Rim
@19	257	3	0.01	0.32	0.3317	2.63	0.04632	1.13	0.4282	291.9	3.2	Rim
@4	257	4	0.02	2.01	0.3570	5.80	0.04633	2.13	0.3676	292.0	6.1	Rim
@17	277	3	0.01	0.14	0.3312	4.42	0.04638	3.98	0.9008	292.3	11.4	Rim
@13	265	4	0.01	0.16	0.3303	2.93	0.04640	2.08	0.7089	292.4	5.9	Rim
@3	364	4	0.01	0.24	0.3390	2.87	0.04672	1.77	0.6176	294.3	5.1	Rim
@5	251	4	0.01	0.69	0.3193	4.68	0.04692	2.12	0.4525	295.6	6.1	Rim
@7	207	3	0.01	1.49	0.3280	5.56	0.04720	2.27	0.4078	297.3	6.6	Rim
@6	170	8	0.05	0.69	0.3318	4.10	0.04783	2.17	0.5306	301.2	6.4	Rim
@1	299	4	0.01	0.17	0.3450	2.72	0.04849	1.93	0.7091	305.3	5.8	Rim
@15	479	5	0.01	0.15	0.3512	2.74	0.04862	2.41	0.8797	306.1	7.2	Rim
* @2*	*451*	*24*	*0.06*	*0.56*	*0.6769*	*2.82*	*0.08389*	*1.08*	*0.3832*	*519.3*	*5.4*	*Core*
Malone Valley–AV16-51 Lws-micaschist												
@8	273	52	0.20	0.61	0.2810	5.20	0.04230	2.80	0.5500	267.0	7.0	Rim
@1	333	9	0.03	0.25	0.3050	3.50	0.04310	2.40	0.6700	272.0	6.0	Rim
@21	168	42	0.26	0.45	0.3222	5.62	0.04312	4.59	0.8165	272.1	12.2	Rim
@10	358	23	0.07	0.20	0.3140	3.50	0.04340	2.50	0.7100	274.0	7.0	Rim
@9	374	7	0.02	0.30	0.2980	3.70	0.04400	2.50	0.6600	277.0	7.0	Rim
@11b	267	54	0.21	0.20	0.3070	4.10	0.04400	2.80	0.6700	278.0	8.0	Rim
@14	308	35	0.12	0.37	0.2960	4.20	0.04430	2.50	0.6000	279.0	7.0	Rim
@24	295	14	0.05	0.62	0.3005	3.82	0.04455	2.08	0.5443	280.9	5.7	Rim
@2	393	15	0.04	0.18	0.3290	3.30	0.04470	2.40	0.7300	282.0	7.0	Rim
@27	236	3	0.01	0.23	0.3205	3.29	0.04493	2.25	0.6837	283.3	6.2	Rim
@28	278	5	0.02	0.45	0.3206	3.00	0.04512	1.15	0.3815	284.5	3.2	Rim
@20	363	43	0.12	0.19	0.3259	2.89	0.04539	2.04	0.7054	286.1	5.7	Rim
@19	200	41	0.21	0.12	0.3323	5.12	0.04540	2.24	0.4370	286.2	6.3	Rim
@15	355	20	0.06	0.49	0.3100	4.00	0.04550	2.30	0.5700	287.0	6.0	Rim
@13	435	56	0.13	0.00	0.3300	3.30	0.04560	2.20	0.6700	287.0	6.0	Rim
@22	275	5	0.02	0.24	0.3190	3.07	0.04560	2.06	0.6694	287.5	5.8	Rim
@7	734	253	0.36	0.07	0.3360	2.40	0.04590	1.90	0.8000	289.0	5.0	Rim
@23	214	7	0.04	0.46	0.3154	4.57	0.04592	3.38	0.7389	289.4	9.6	Rim
@12	192	23	0.12	0.37	0.3270	5.10	0.04610	3.00	0.5900	290.0	9.0	Rim
@16b	230	2	0.01	0.00	0.3390	3.37	0.04626	2.16	0.6395	291.5	6.1	Rim
@26	418	59	0.15	0.25	0.3246	2.66	0.04640	1.82	0.6824	292.4	5.2	Rim
@6	167	49	0.30	0.81	0.3250	6.60	0.04660	3.20	0.4800	294.0	9.0	Rim
@18	210	14	0.07	0.26	0.3362	3.48	0.04709	2.27	0.6516	296.6	6.6	Rim
@25	333	18	0.06	0.25	0.3261	3.55	0.04712	2.86	0.8062	296.8	8.3	Rim
@17	339	16	0.05	0.48	0.3195	3.25	0.04720	1.87	0.5753	297.3	5.4	Rim
* @3*	*322*	*92*	*0.30*	*0.08*	*0.4000*	*3.30*	*0.05520*	*2.50*	*0.7400*	*346.0*	*8.0*	*Core*
Monte Mucrone–AV17-07 micaschist												
* @5*	*119*	*9*	*0.08*	*1.33*	*0.1364*	*6.14*	*0.01974*	*2.89*	*0.4701*	*126.0*	*3.6*	*Possible Pb loss*
* @7*	*348*	*17*	*0.05*	*1.13*	*0.1573*	*6.50*	*0.02366*	*2.48*	*0.3815*	*150.7*	*3.7*	*Possible Pb loss*
* @26*	*330*	*2*	*0.01*	*0.04*	*0.1769*	*12.10*	*0.02413*	*12.00*	*0.9922*	*153.7*	*18.2*	*Possible Pb loss*
* @6*	*271*	*2*	*0.01*	*0.28*	*0.1933*	*3.26*	*0.02697*	*2.05*	*0.6295*	*171.5*	*3.5*	*Possible Pb loss*
* @9*	*345*	*5*	*0.01*	*0.51*	*0.2143*	*3.37*	*0.03078*	*1.82*	*0.5410*	*195.4*	*3.5*	*Possible Pb loss*
* @29*	*266*	*3*	*0.01*	*0.25*	*0.2488*	*2.92*	*0.03301*	*1.91*	*0.6541*	*209.4*	*3.9*	*Possible Pb loss*
* @11*	*243*	*2*	*0.01*	*0.14*	*0.2394*	*3.05*	*0.03308*	*2.01*	*0.6577*	*209.8*	*4.2*	*Possible Pb loss*
* @2*	*392*	*2*	*0.00*	*0.36*	*0.2313*	*6.35*	*0.03412*	*5.82*	*0.9161*	*216.3*	*12.4*	*Possible Pb loss*
* @32*	*221*	*4*	*0.02*	*0.43*	*0.2771*	*4.43*	*0.03800*	*2.06*	*0.4649*	*240.4*	*4.9*	*Possible Pb loss*
* @31*	*332*	*8*	*0.03*	*0.47*	*0.2709*	*3.21*	*0.03900*	*1.79*	*0.5586*	*246.6*	*4.3*	*Possible Pb loss*
* @3*	*220*	*3*	*0.01*	*0.29*	*0.2731*	*3.41*	*0.03958*	*2.13*	*0.6252*	*250.2*	*5.2*	*Possible Pb loss*
* @12*	*285*	*3*	*0.01*	*0.43*	*0.2856*	*3.87*	*0.04132*	*1.97*	*0.5093*	*261.0*	*5.0*	*Possible Pb loss*
* @27*	*45*	*2*	*0.04*	*1.00*	*0.2768*	*6.05*	*0.04289*	*1.15*	*0.1894*	*270.7*	*3.0*	*Possible Pb loss*
* @10*	*507*	*5*	*0.01*	*0.91*	*0.3026*	*3.48*	*0.04328*	*1.64*	*0.4720*	*273.1*	*4.4*	*Possible Pb loss*
@22	291	3	0.01	1.79	0.2968	5.59	0.04461	1.91	0.3411	281.3	5.3	Rim1
@20	178	3	0.02	0.12	0.3194	2.86	0.04512	2.53	0.8831	284.5	7.0	Rim1
@17	232	5	0.02	0.25	0.3097	3.02	0.04558	1.91	0.6321	287.3	5.4	Rim1
@19	1010	12	0.01	0.38	0.4202	7.81	0.04624	3.00	0.3843	291.4	5.5	Rim1
@4	279	4	0.01	0.95	0.3347	3.99	0.04624	1.92	0.4814	291.7	5.5	Rim1
@24	211	6	0.03	2.55	0.3743	14.47	0.04629	3.70	0.2554	292.9	10.6	Rim1
@8	196	4	0.02	0.62	0.3232	4.16	0.04649	2.19	0.5257	293.4	6.3	Rim1
@30	237	6	0.03	0.68	0.3558	14.32	0.04657	1.90	0.1324	296.9	5.5	Rim1
* @33*	*239*	*62*	*0.27*	*1.41*	*0.5544*	*4.05*	*0.04714*	*2.03*	*0.5027*	*419.8*	*8.3*	*Core*
* @35*	*205*	*98*	*0.49*	*0.21*	*0.5560*	*2.68*	*0.06730*	*2.05*	*0.7644*	*445.5*	*8.8*	*Core*
* @21*	*370*	*107*	*0.30*	*0.04*	*0.5671*	*1.97*	*0.07155*	*1.67*	*0.8477*	*451.5*	*7.3*	*Core*
* @16*	*229*	*123*	*0.55*	*0.07*	*0.5864*	*2.44*	*0.07256*	*2.02*	*0.8283*	*463.3*	*9.0*	*Core*
Monte Mucrone–AV17-16 eclogite												
* @6*	*12*	*1*	*0.06*	*2.21*	*0.2901*	*25.77*	*0.03508*	*8.02*	*0.3113*	*222.3*	*17.5*	*Possible Pb loss*
* @7b*	*48*	*16*	*0.34*	*0.00*	*0.2588*	*9.14*	*0.03689*	*8.00*	*0.8751*	*233.5*	*18.3*	*Possible Pb loss*
@4	43	14	0.33	0.29	0.3114	6.91	0.04214	4.13	0.5974	266.1	10.8	Core
@11	16	4	0.27	2.29	0.2535	26.99	0.04236	7.21	0.2671	267.4	18.9	Core
@12	41	13	0.33	1.76	0.2713	15.17	0.04270	4.21	0.2776	269.5	11.1	Core
@9	289	48	0.17	0.26	0.3281	3.76	0.04357	2.75	0.7306	275.0	7.4	Core
* @9*	*50*	*20*	*0.42*	*0.58*	*0.3406*	*9.73*	*0.04772*	*3.93*	*0.4039*	*300.5*	*11.5*	*Possible inheritance*
* @10*	*78*	*42*	*0.56*	*1.06*	*0.4848*	*6.35*	*0.06240*	*3.20*	*0.5046*	*390.2*	*12.1*	*Possible inheritance*

**Fig. 6:**
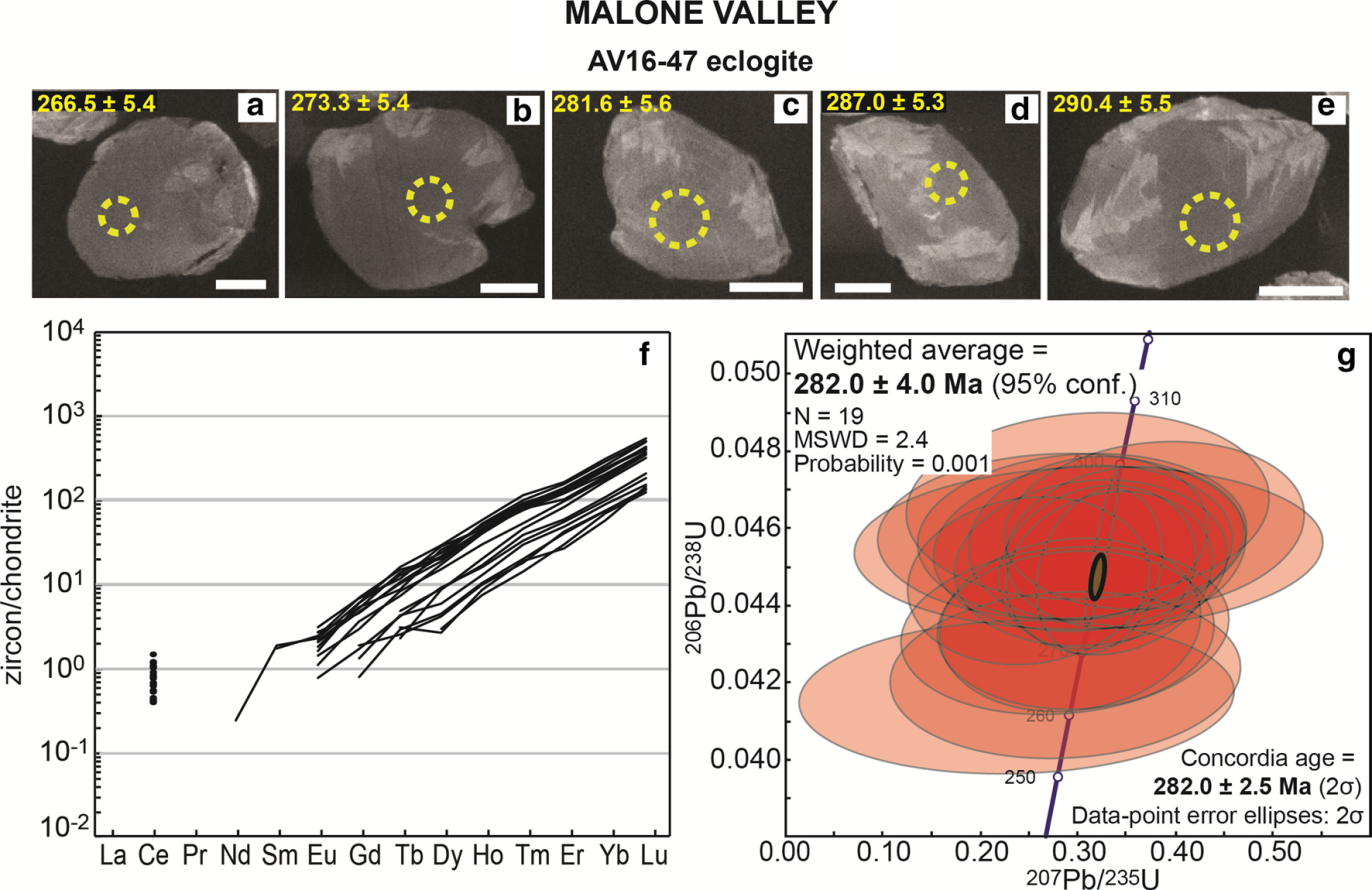
Zircon in eclogite AV16-47 from Malone Valley. **a**–**e** Selected CC images of analysed zircon grains. Scale bar in all images is 30 μm. Measured spots are shown (yellow circles) with the associated date (± 1σ Ma). **f** REE patterns of zircons. Permian zircon crystals interpreted as magmatic have a steep REE pattern. **g** Concordia plot of zircon dates and weighted average ^206^Pb/^238^U age. The green circle represents the calculated Concordia age

Data were processed using SQUID 2.50. The dates that were not used for the age calculations shown in Figs. 3, 6 and 10 are reported in italics

^a^% of common ^206^Pb on total ^206^Pb

Allanite grains are typically elongated in the direction of the foliation, with a length up to 100 μm and a width of 20–30 μm. In BSE they have a brighter core sometimes showing an internal zoning, and darker rims growing preferentially in the direction of the foliation (Fig. 4). Th/U ratio ranges between 5.6 and 20.0. On the TW diagram, the allanite U–Pb analyses concentrate in a cluster due to the limited variation in the total ^207^Pb/^206^Pb and ^238^U/^206^Pb (Table 3). The regression yields an initial ^207^Pb/^206^Pb of 0.86 ± 0.02 (2σ), which is comparable to the expected model common Pb at 65 Ma (Stacey and Kramers 1975). Due to the high per cent of initial Pb in the analyses (94–96%) and the limited variation in the total ^238^U/^206^Pb among individual analyses, no intercept age could be obtained for this sample with a regression in the TW diagram.

#### Lws-micaschist AV16-51

Zircon grains recovered from this sample are characterized by the presence of rounded cores (one concordant analysis at 346 ± 8 Ma) with vary variable zoning patterns overgrown by a light grey, unzoned rim of maximum width of 20–30 μm (Fig. 3) with a large variation in Th/U (0.01–0.36) (Table 4). The rim U–Pb analyses form a tight cluster on the Concordia diagram with an early Permian average age of 285.9 ± 2.9 Ma. REE patterns of zircon rim show enrichment of HREE with respect to MREE (Gd_N_/Lu_N_ = 0.01–0.13, Fig. 3 and Additional file 4) and a negative Eu anomaly (Eu/Eu* = 0.11–0.51, one analysis Eu/Eu* = 0.67). Titanium content is typically between 1.8 and 20 µg/g (Additional file 4). The calculated Ti-in-zircon temperature values scatter between ~ 610 and ~ 835 °C (Additional file 5). However, this must be interpreted as a minimum estimate as the buffering Ti phase in the Permian assemblage is unknown (titanite is present in the HP assemblage and contains no rutile relic).

#### Eclogite AV16-47

Zircon grains are rounded and characterised by internal sector and fir-tree zoning (Fig. 6). No significant resorption and no overgrowth are observed. Uranium and Th contents are low (6–16 and 1–6 µg/g, respectively) as typical of mafic rocks. The Th/U ratio ranges between 0.11 and 0.44 (Table 2). The ^206^Pb/^238^U dates span from 263 to 293 Ma and result in a weighted age of 282.0 ± 4.0 Ma with excess scatter (MSWD = 2.4). The REE pattern of the zircon is characterized by a steep increase from mid to HREE (Gd_N_/Lu_N_ = 0.01–0.02, Fig. 6 and Additional file 4). Titanium concentrations are low (1.2–3.3 µg/g) and the lack of quartz in the rock prevents the calculation of meaningful Ti-in-zircon temperatures.

### Ivozio Complex

#### Eclogite AV16-21

**Fig. 7:**
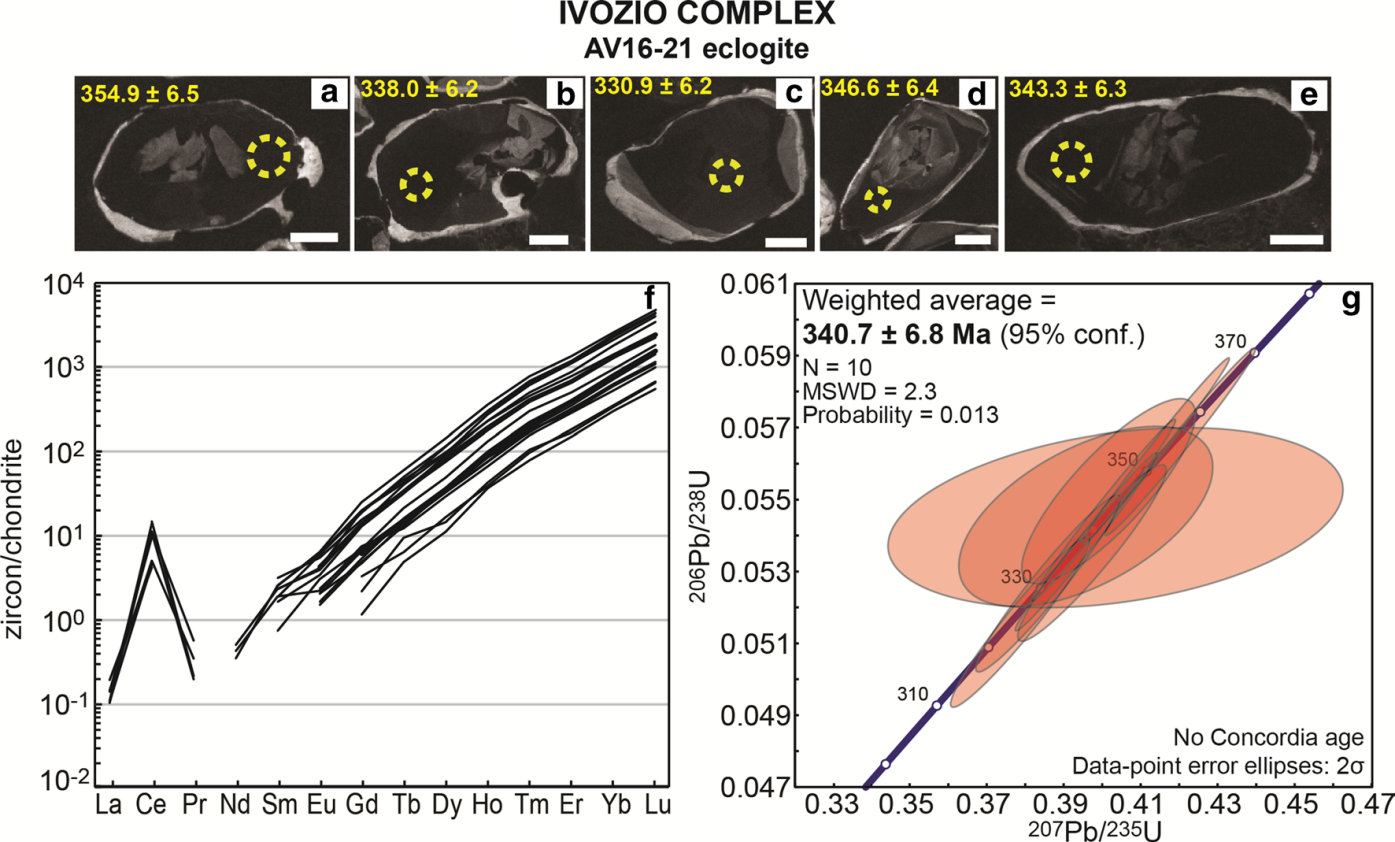
Eclogite AV16-21, Ivozio Complex. **a**–**e** Selected CC images of analysed zircon grains. Scale bar in all images is 30 μm. Measured spots are shown (yellow circles) with the associated date (± 1σ Ma). **f** REE patterns of zircon cores. **g** Concordia plot of zircon core dates and weighted average ^206^Pb/^238^U age

Only a few zircon grains could be recovered from eclogite AV16-21. They are characterized by CC-dark cores with weak oscillatory zoning. CC-bright rims are typically < 10 μm thick (Fig. 7) and thus U–Pb and trace element analysis was focused on the characterization of the zircon cores. In the dated cores, Th/U ratio ranges between 0.03–0.19 (Table 2). Zircon cores yield an early Carboniferous average age of 340.7 ± 6.8 Ma (Fig. 7, weighted average plots are shown in Additional file 3). Older dates were obtained in three cores and indicate the presence of inherited components.

Trace element patterns of zircon cores show a steep increase in REE with increasing atomic number, a strong Ce positive anomaly and a very weak Eu anomaly (Fig. 7f, Additional file 4). The few thin rims could not be analysed for trace elements. Zircon cores contain 2–7 µg/g of Ti and the Ti-in-zircon thermometer (Watson et al. 2006) returns an avergae temperature of 663 ± 18 °C (1σ). However, this has to be interpreted as a minimum temperature because it is impossible to establish what the Ti-buffering phase was; the rutile present in the rock is interpreted to be part of the later Alpine HP mineral assemblage.

#### Blueschist AV16-53

**Fig. 8:**
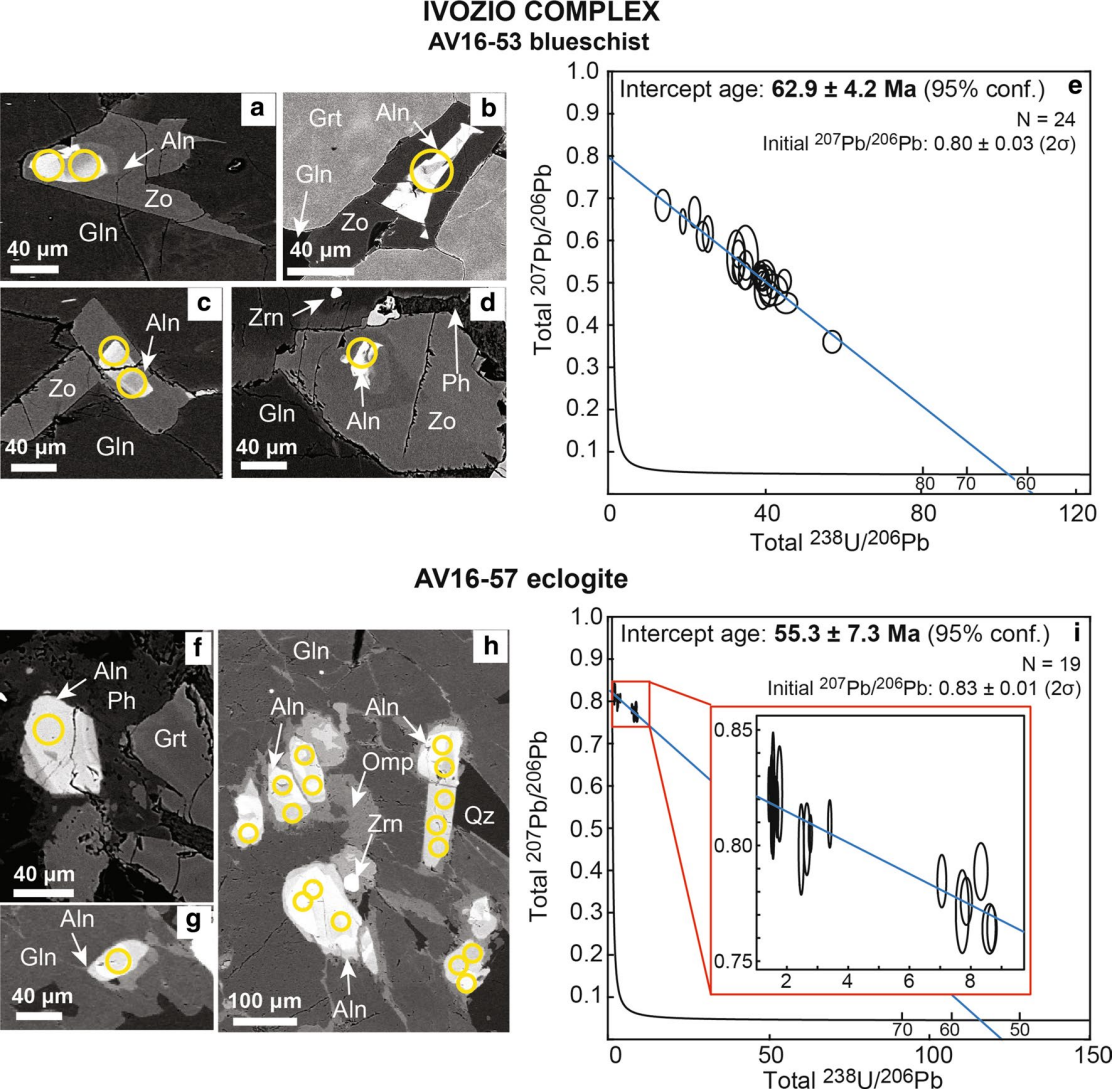
Allanite in samples from Ivozio Complex. Blueschist AV16-53: **a**–**d** Thin section BSE images of zoned allanite (Aln) with a zoisite rim (Zo), associated to glaucophane (Gln) and, to a minor extent, to garnet (Grt) and phengite (Ph). Zircon (Zrn) < 10 μm in size are present. Yellow circles represent analysed spots. **e** Tera-Wasserburg plot for LA-ICP-MS U–Pb allanite analyses. Data-point error ellipses are 2σ. Eclogite AV16-57: **f**–**h** Thin section BSE images of zoned allanite (Aln) associated to glaucophane (Gln) and phengite (Ph). Garnet (Grt) is fractured and partially resorbed. Quartz (Qz) and μm-size zircons (Zrn) are also present. Yellow circles represent analysed spots. **i** Tera-Wasserburg plot for LA-ICP-MS U–Pb allanite analyses. Data-point error ellipses are 2σ

Allanite in the blueschist AV16-53 is found mostly in contact with glaucophane, often as inclusion, and to a lesser extent in contact with garnet and phengite (Fig. 8). Allanite cores are brighter in BSE images and are surrounded by a darker, euhedral zoisitic rim. The Th/U ratio ranges between 0.4 and 8.3 (Table 5). The regression of the U–Pb analyses in a TW diagram defines a lower intercept age of 62.9 ± 4.2 Ma with an initial ^207^Pb/^206^Pb of 0.80 ± 0.03 (2σ), which is slightly lower than the model common Pb at 65 Ma (0.84, Stacey and Kramers 1975).

**Table 5:** U, Th and Pb LA-ICP-MS allanite data for the Ivozio Complex samples

Spot name	Th/U	f206^a^	f208^b^	^238^U/^206^Pb total	± 1σ%	^208^Pb/^232^Th total	± 1σ%	^207^Pb/^206^Pb total	± 1σ%	^206^Pb/^208^Pb total	± 1σ%
AV16-53 blueschist											
c7	1.4	0.47	0.94	45.3842	2.67	0.02610	2.23	0.3975	4.17	0.9643	3.06
c8	3.2	0.68	0.92	32.5109	3.58	0.02347	2.16	0.5616	5.61	0.6415	3.32
c9	1.0	0.83	1.00	21.9071	3.38	0.11706	2.48	0.6679	2.72	0.6246	2.47
c10	1.6	0.76	1.00	25.2766	2.62	0.05869	2.57	0.6163	3.46	0.6552	3.08
c11	0.9	0.58	1.00	39.3254	2.70	0.04890	2.28	0.4830	4.60	0.8628	3.77
c12	0.9	0.62	1.00	39.3052	2.04	0.05599	1.87	0.5100	2.76	0.7757	2.37
c13	1.5	0.62	0.95	38.9272	1.78	0.03575	1.52	0.5144	2.03	0.7335	1.73
c14	2.9	0.62	0.90	39.7005	2.59	0.01937	2.45	0.5135	2.89	0.6944	2.16
c15	2.6	0.62	0.93	34.7896	2.30	0.02448	1.80	0.5167	3.05	0.7163	2.22
c16	3.2	0.62	0.88	39.9085	3.27	0.03187	3.74	0.5124	4.06	0.6841	3.41
d8	1.8	0.66	0.99	34.9907	2.60	0.03625	3.94	0.5421	3.18	0.7221	2.73
d9	3.2	0.85	1.00	13.7847	7.00	0.06560	7.83	0.6840	2.72	0.5769	2.84
d10	1.2	0.58	1.00	42.0500	4.27	0.03704	3.98	0.4842	3.76	0.8437	3.48
d12	2.7	0.61	0.95	44.8335	1.90	0.01756	3.59	0.5054	2.62	0.7490	2.06
d13	2.6	0.68	0.93	34.8697	4.60	0.02557	4.20	0.5608	6.72	0.6537	3.77
d16	1.1	0.80	1.00	18.9065	2.11	0.12460	3.75	0.6460	2.27	0.6029	1.98
e7	8.3	0.64	0.72	33.0024	2.19	0.01054	3.11	0.5321	2.69	0.5349	2.03
e8	1.8	0.59	0.91	40.8419	3.07	0.02757	3.32	0.4883	4.44	0.7411	2.85
e9	1.1	0.58	0.96	41.6844	1.51	0.03998	3.13	0.4831	1.99	0.7945	1.78
e10	2.6	0.75	0.96	23.9594	3.25	0.03838	4.04	0.6111	2.82	0.6153	2.67
e11	1.0	0.64	1.00	38.6649	1.95	0.05483	3.16	0.5297	2.04	0.7533	2.10
e14	0.4	0.62	1.00	38.6961	2.46	0.12957	3.51	0.5133	2.80	0.8059	2.11
e15	1.5	0.42	0.83	57.0535	2.05	0.01856	3.24	0.3610	3.62	0.9548	2.31
e16	1.7	0.69	0.97	33.0640	2.37	0.03963	3.39	0.5691	2.81	0.6672	2.75
AV16-57 eclogite											
b7	7.7	0.97	0.98	2.6549	1.95	0.1854	1.99	0.8027	0.97	0.4844	0.93
b8	16.7	0.97	0.97	1.5166	1.32	0.1516	2.03	0.8092	0.79	0.4753	0.71
b9	1.1	0.95	1.00	8.3277	1.32	0.3687	2.27	0.7887	0.78	0.5034	0.71
b10	16.7	0.99	0.98	1.5057	1.37	0.1488	2.08	0.8240	0.92	0.4741	0.85
b11	20.0	0.99	0.97	1.6376	1.20	0.1140	1.95	0.8198	0.62	0.4709	1.20
b12	0.7	0.92	0.99	8.6096	1.37	0.5450	2.02	0.7640	0.70	0.5161	0.61
b13	5.9	0.99	1.00	1.7627	2.53	0.3579	2.05	0.8223	1.25	0.4835	2.53
b14	2.0	0.97	1.00	2.7664	1.02	0.6386	2.20	0.8051	0.50	0.4936	0.51
b15	8.3	0.98	0.99	1.6850	1.21	0.2575	2.08	0.8156	0.75	0.4832	1.21
b16	12.5	0.99	0.99	1.4259	1.15	0.1937	2.14	0.8221	0.71	0.4770	0.63
c7	16.7	0.99	1.00	1.5522	1.55	0.1356	1.62	0.8215	1.67	0.4836	1.55
c8	1.5	0.97	0.99	3.4007	0.85	0.7082	1.60	0.8093	0.63	0.4882	0.58
c9	1.0	0.93	0.98	7.7205	1.37	0.4574	1.71	0.7714	1.16	0.5089	1.20
c10	0.7	0.92	1.00	8.6707	0.80	0.5316	1.54	0.7643	0.65	0.5217	0.66
c11	0.8	0.93	1.00	7.8524	1.18	0.5374	1.66	0.7759	0.66	0.5141	1.18
c12	4.3	0.99	0.99	1.5587	1.22	0.5318	1.64	0.8186	0.66	0.4828	0.73
c13	6.3	0.96	0.97	2.4700	1.75	0.2223	1.77	0.7983	1.23	0.4841	1.36
c14	0.7	0.94	1.00	7.0530	1.04	0.6764	1.55	0.7846	0.73	0.5067	0.69
c16	16.7	0.99	0.98	1.4934	0.98	0.1405	1.55	0.8222	0.76	0.4732	0.68

^a,b^See Table 3

#### Eclogite AV16-57

Allanite in the eclogite AV16-57 is found only in correspondence of the domains enriched in glaucophane and phengite, while it is absent in the rest of the rock. The BSE images show allanite cores that are brighter than allanite rims (Fig. 8). The Th/U ratio is very variable, between 0.7 and 16.7 (Table 5). The U–Pb analyses are relatively rich in initial Pb and thus their regression in a TW diagram defines an age with a relatively large uncertainty of 55.3 ± 7.3 Ma. The regression defines an initial ^207^Pb/^206^Pb of 0.83 ± 0.01 (2σ), which corresponds within uncertainty to the model common Pb at 65 Ma (0.84, Stacey and Kramers 1975).

### Chiusella Valley

#### Mn-rich quartzite VC10-04

**Fig. 9:**
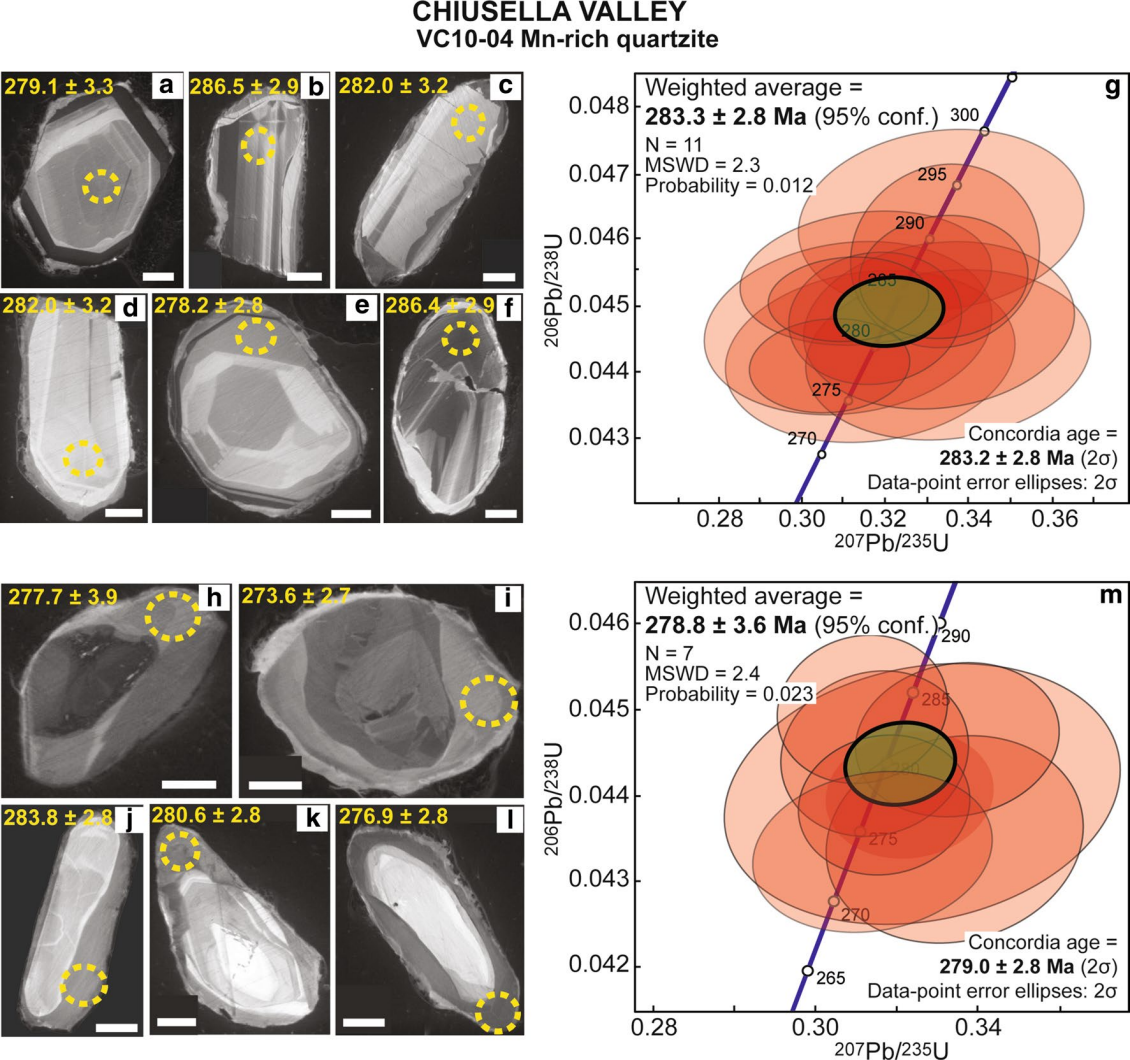
Zircon in Mn-rich quartzite VC10-04 from Chiusella Valley. **a**–**f** Selected CC images of analysed zircon cores. Scale bar in all images is 30 μm. Measured spots are shown (yellow circles) with the associated date (± 1σ Ma). **g** Concordia plot of zircon core dates and weighted average ^206^Pb/^238^U age. **h**–**l** Selected CC images of analysed zircon rim1. Scale bar in all images is 30 μm. Measured spots are shown (yellow circles) with the associated date (± 1σ Ma). **m** Concordia plot of zircon rim1 dates and weighted average ^206^Pb/^238^U age

Zircon grains in this sample are characterized by the presence of cores generally displaying oscillatory or sector zoning, occasionally partially resorbed and overgrown by one or two unzoned, discontinuous rims (Fig. 9). The rim1 is dark grey and has a thickness up to 30 μm; rim2 is slightly lighter in colour in the CC images, up to 5 μm thick, and therefore dating was focused on core and rim1. Zircon cores and rim1, while different in morphology, cannot be distinguished on the basis of their U–Pb dates. Zircon core dates span between 291.8 ± 3.5 Ma and 261.6 ± 4.8 Ma (Table 6); the group of 11 older dates yield an average age of 283.2 ± 2.8 Ma. Zircon rim1 vary in age between 281.6 ± 2.8 Ma and 274.6 ± 6.2 Ma (Table 6) with an average of 278.8 ± 3.6 Ma (Fig. 9, weighted average plots are shown in Additional file 3).

**Table 6:** U and Pb LA-ICP-MS zircon data of the Mn-rich quartzite VC10-04 from Chiusella Valley

Spot name	Concordia diagram					^206^Pb/^238^U Age (Ma)	± 1σ	Comments
	^207^Pb/^235^U	± 1σ %	^206^Pb/^238^U	± 1σ %	ρ			
Chiusella Valley–VC10-04 Mn-rich quartzite								
04mrc11	0.316	3.813	0.043	0.884	0.15	273.6	2.7	Rim1
04mrc13	0.334	3.850	0.043	1.134	0.12	274.4	3.1	Rim1
03mrb06	0.317	2.707	0.044	0.758	0.08	276.9	2.8	Rim1
03mrc11	0.323	2.601	0.044	0.689	0.09	277.7	3.9	Rim1
03mrc07	0.315	2.987	0.044	0.884	0.10	280.6	2.8	Rim1
03mrd12	0.335	3.044	0.045	0.820	0.11	281.6	2.8	Rim1
04mrb11	0.312	2.735	0.045	0.782	0.09	283.8	2.8	Rim1
* 03mrc06*	*0.306*	*6.537*	*0.041*	*1.846*	*0.28*	*261.6*	*4.8*	*Core*
* 04mrc14*	*0.289*	*4.820*	*0.042*	*1.465*	*0.14*	*263.7*	*3.9*	*Core*
* 03mrd06*	*0.309*	*2.542*	*0.043*	*1.010*	*0.07*	*268.3*	*2.7*	*Core*
* 03mrd11*	*0.320*	*2.020*	*0.043*	*0.904*	*0.03*	*273.8*	*2.7*	*Core*
* 04mrb07*	*0.315*	*3.365*	*0.044*	*0.836*	*0.13*	*275.0*	*2.8*	*Core*
* 03mrb07*	*0.320*	*2.652*	*0.044*	*0.828*	*0.06*	*275.5*	*2.8*	*Core*
* 03mrd07*	*0.314*	*2.519*	*0.044*	*1.086*	*0.05*	*276.7*	*3.0*	*Core*
* 04mrc12*	*0.316*	*3.701*	*0.044*	*0.875*	*0.14*	*276.8*	*2.8*	*Core*
* 04mrc06*	*0.326*	*6.074*	*0.044*	*1.408*	*0.18*	*277.7*	*2.8*	*Core*
04mrb13	0.307	2.570	0.044	0.656	0.09	278.2	2.8	Core
03mrb05	0.316	4.493	0.044	1.195	0.14	279.1	3.3	Core
03mrc10	0.335	4.174	0.044	1.182	0.14	279.3	3.3	Core
04mrc05	0.311	4.540	0.045	1.191	0.15	281.7	3.4	Core
03mrc12	0.333	3.994	0.045	1.151	0.13	282.0	3.2	Core
04mrb05	0.317	2.670	0.045	0.907	0.08	282.6	2.8	Core
04mrb12	0.311	2.530	0.045	0.576	0.09	284.4	2.8	Core
04mrc07	0.315	3.913	0.045	0.908	0.15	286.4	2.9	Core
04mrb06	0.334	2.480	0.045	0.833	0.07	286.5	2.9	Core
03mrd05	0.334	2.777	0.046	1.233	0.07	288.5	3.6	Core
03mrc05	0.333	4.204	0.046	1.205	0.13	291.8	3.5	Core

Data were processed using Lamtool. The dates that were not used for the age calculations shown in Fig. 9 are reported in italics

### Monte Mucrone

#### Micaschist AV17-07

**Fig. 10:**
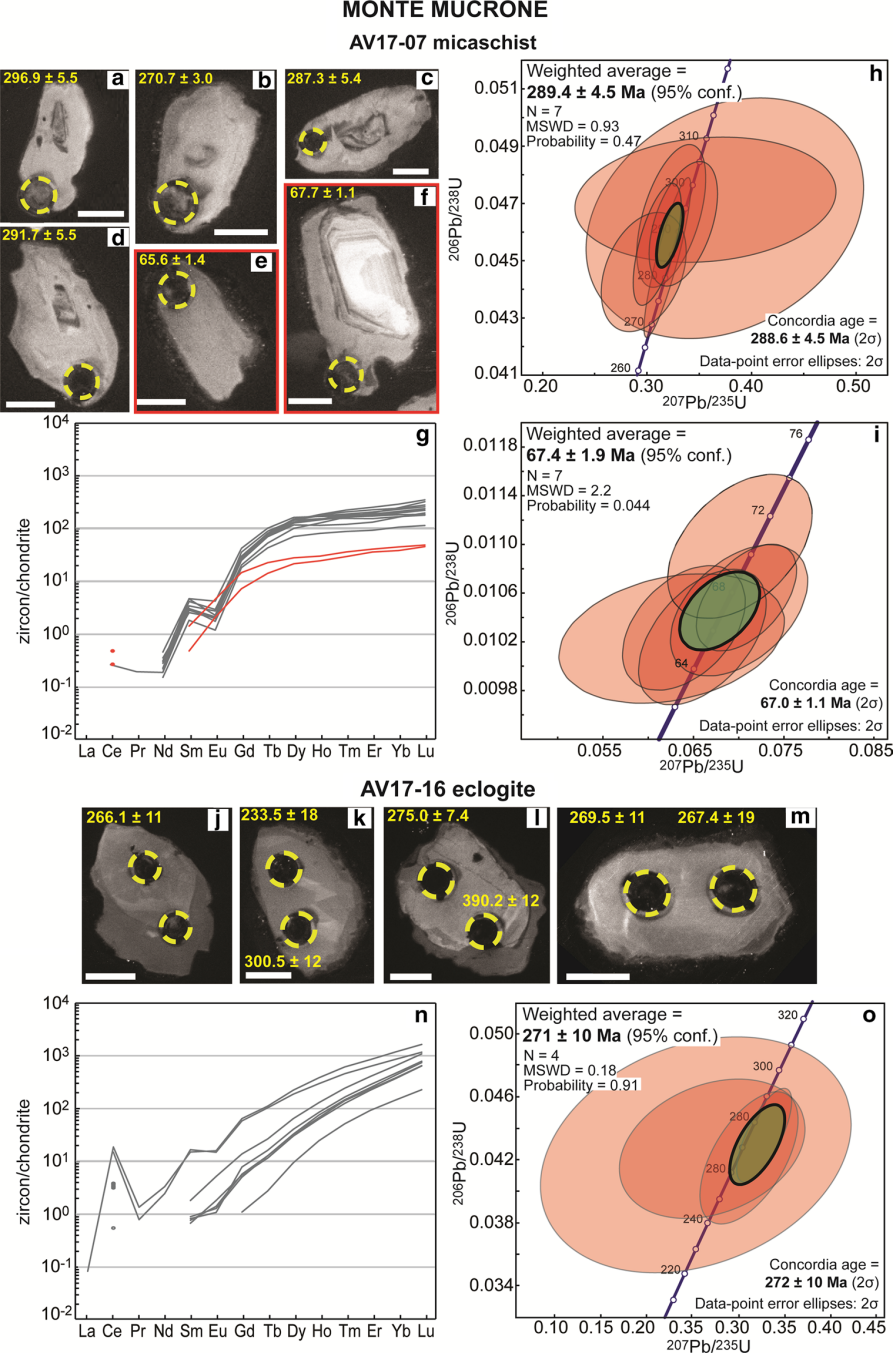
Zircon in samples from Monte Mucrone. Micaschist AV17-07: **a**–**f** Selected CC images of analysed zircon grains. Scale bar in all images is 30 μm. Measured spots are shown (yellow circles) with the associated date (± 1σ Ma). The two grains yielding Alpine ages are marked by a red frame. **g** REE patterns of zircon domains of Permian age (rim1, grey lines) and of Alpine Alpine age (rim2, red lines). **h** Concordia plot for zircon rim1 and weighted average ^206^Pb/^238^U age. The green circle represents the calculated Concordia age. **i** Concordia plot for zircon rim2 and weighted average ^206^Pb/^238^U age. The green circle represents the calculated Concordia age. Eclogite AV17-16: **j**–**m** CC images of the recovered and analysed zircon grains. Scale bar in all images is 30 μm. Measured spots are shown (yellow circles) with the associated date (± 1σ Ma). **n** REE patterns of zircon cores. **o** Concordia plot for zircon core and weighted average ^206^Pb/^238^U age. The green circle represents the calculated Concordia age

Zircon grains in this sample contain partially resorbed cores with oscillatory zoning that are overgrown by one or two unzoned rims (Fig. 10). The two rims are distinct in composition and age, but hardly distinguishable in the CC images. Zircon rim1 has a low Th/U of 0.01–0.03 and yield an age of 289.4 ± 4.5 Ma (Table 4). Rim1 analyses are characterized by a flat M- to HREE pattern (Gd_N_/Lu_N_ = 0.06–0.30) and a moderate Eu negative anomaly (Eu/Eu* = 0.11–0.27, Fig. 10 and Additional file 4). Rim1 contains variable Ti (1.4–12.8 µg/g) and Ti-in-zircon temperature values scatter between ~ 610 and ~ 755 °C. However, all rutile in the sample may be Alpine, and this temperature must be considered as a minimum value.

Zircon rim2 has a low Th/U ratio of ≤ 0.01 and a late Cretaceous age of 67.4 ± 1.9 Ma (Table 7, Fig. 10). This external rim has lower HREE contents with respect to rim1, a flat HREE pattern (Gd_N_/Lu_N_ = 0.16–0.30) and no Eu anomaly (Eu/Eu* = 1.00–1.12, Fig. 10 and Additional file 4). Titanium content for the two analyses is 1.0 and 2.0 µg/g, yielding temperatures of ~ 575 and ~ 615 °C (Additional file 5).

**Table 7:** U, Th and Pb SIMS zircon data of the micaschist AV17-07 from Monte Mucrone that yield the age of the Alpine HP metamorphic event

Spot name	U (µg/g)	Th (µg/g)	Th/U	f206^a^	Concordia diagram (^208^Pb corr.)					^206^Pb/^238^U Age (Ma)	± 1σ	Comments
					^207^Pb/^235^U	± 1σ%	^206^Pb/^238^U	± 1σ%	ρ			
Monte Mucrone–AV17-07 micaschist												
@13	207	1	0.01	2.58	0.06260	8.13	0.01023	2.12	0.2629	65.6	1.4	Rim2
@18	199	1	0.01	2.25	0.06597	4.35	0.01024	2.22	0.4904	65.7	1.5	Rim2
@1	241	2	0.01	1.72	0.06392	4.32	0.01032	2.02	0.4221	66.2	1.3	Rim2
@25	198	1	0.01	2.05	0.07004	4.43	0.01046	2.11	0.4485	67.1	1.4	Rim2
@34	339	2	0.01	0.69	0.07086	3.19	0.01056	1.70	0.5095	67.7	1.1	Rim2
@14	178	1	0.01	2.32	0.07012	4.65	0.01098	2.19	0.4475	70.4	1.5	Rim2
@23	287	1	0.01	0.36	0.07005	4.27	0.01124	2.89	0.6531	72.0	2.1	Rim2

Data were processed using SQUID 2.50

^a^% of common ^206^Pb on total ^206^Pb

#### Eclogite AV17-16

This eclogite sample contains few, small (diameter of 50–80 μm) anhedral zircon crystals with lobate boundaries, that display a brighter core in CC images and a darker, discontinuous rim (Fig. 10). Th/U ratio in both cores and rims is between 0.06 and 0.56. Dating results are scattered between 390 and 222 Ma, with four U–Pb analyses concordant at 272 ± 10 Ma (Table 4).

Light REE show a pronounced positive Ce-anomaly (only two analyses had LREE detectable) and a slight negative Eu anomaly (Eu/Eu* = 0.25–0.66, Fig. 10, Additional file 4). All the analyses display a steep HREE enrichment with respect to MREE; the Gd_N_/Lu_N_ value increases with decreasing REE content (Gd_N_/Lu_N_ = 0.05–0.01).

## Discussion

### Carboniferous magmatism

Zircon cores in eclogite AV16-21 from the Ivozio Complex yield an age of 340.7 ± 6.8 Ma. According to the steep REE pattern, the plain zoning and the relatively high Th/U ratio (0.03–0.19), these zircon cores are interpreted as magmatic, therefore yielding a crystallization age for the Ivozio Complex gabbro. The calculated Ti-in-zircon temperature (663 ± 18 °C) is too low to reflect the solidus of the gabbro. Zircon from metamorphosed mafic rocks has been reported to have low Ti contents (e.g. 15–25 µg/g of Ti in magmatic zircon for metagabbros from the Lanzo massif, Kaczmarek et al. 2008), while higher Ti contents (up to 100 µg/g, resulting in temperatures up to ~ 1000 °C) have been described in zircon from not metamorphosed gabbros in oceanic crust (Grimes et al. 2009). This anomaly might be due to Ti loss during metamorphism, but further investigations are required to clarify this issue. The lack of documentation on zircon trace element in continental gabbros prevents any comparison with data from gabbro emplaced in a tectonic context similar to that of the SZ.

**Fig. 11:**
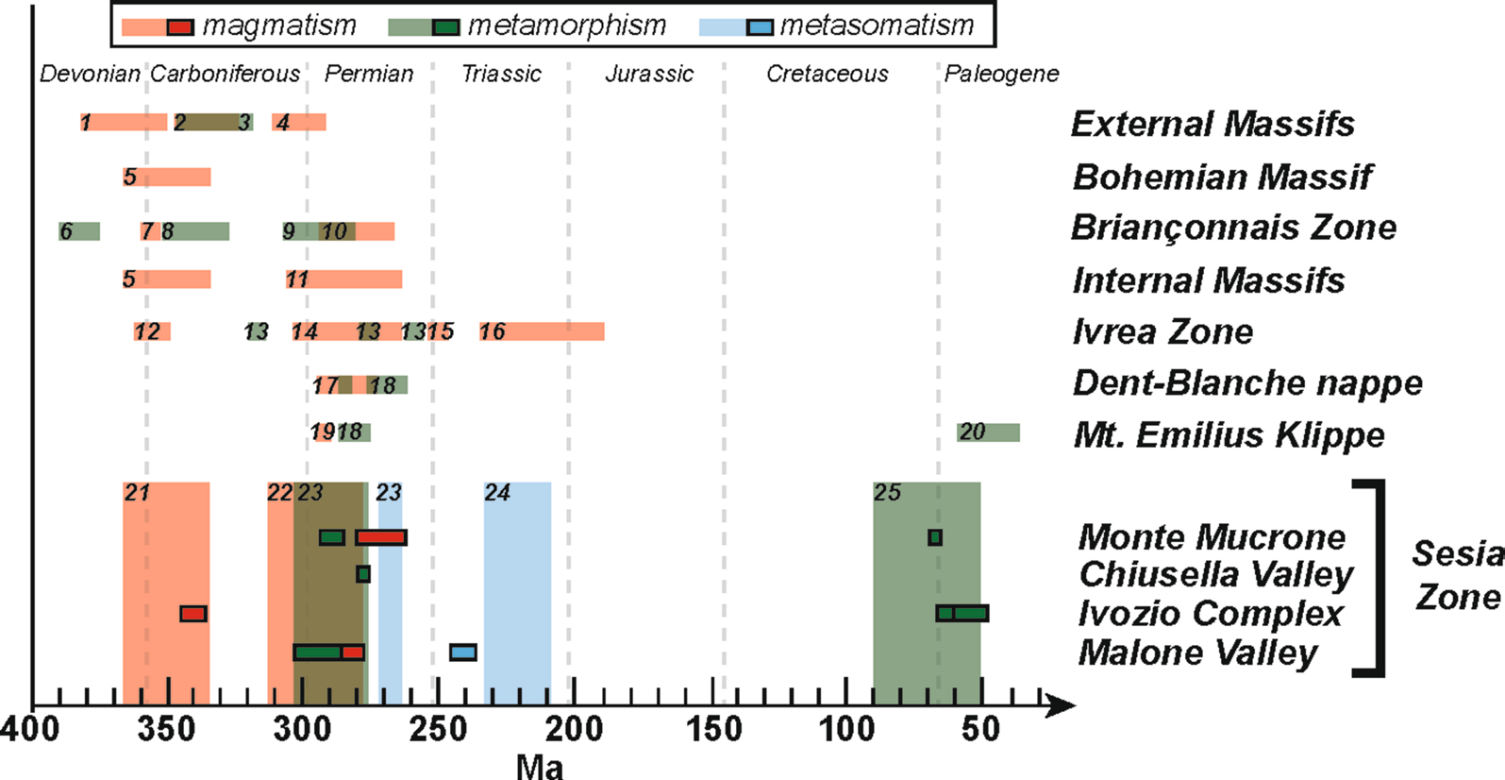
Summary of the magmatic and metamorphic ages in the SZ compared with literature data from relevant units of the Western Alps, the Ivrea Zone and various portions of the Variscan chain. Filled boxes represent age data from this study; semi-transparent boxes represent literature ages: (1) Ménot et al. (1988); (2) Schaltegger and Corfu (1992), Bussy et al. (2000), Guerrot and Debon (2000), Rubatto et al. (2001), Musumeci and Colombo (2002), Oliot et al. (2015); (3) Bussy et al. (2000); Rubatto et al. (2001, 2010); (4) Schaltegger and Corfu (1992), Schaltegger (1993), Bussy and von Raumer (1994), Schaltegger and Corfu (1995), Bussy et al. (2000), Capuzzo and Bussy (2000), Guerrot and Debon (2000), Bussien Grosjean et al. (2017); (5) Pin et al. (1988); (6) Giacomini et al (2007); (7) Bertrand et al. (2000), Guillot et al. (2012); (8) Monié (1990), Bussy et al. (1996); (9) Giorgis et al. (1999); (10) Bussy et al. (1996), Scheiber et al. (2014); (11) Bussy and Cadoppi (1996), Paquette et al. (1999), Bertrand et al. (2000), Liati et al. (2001), Pawlig (2001), Bertrand et al. (2005), Ring et al. (2005); (12) Vavra et al. (1996); (13) Ewing et al. (2013); (14) Pin (1986), Voshage et al. (1987), Mayer et al. (2000), Garuti et al. (2001), Vavra and Schaltegger (1999), Peressini et al. (2007), Karakas et al. (2019); (15) Wright and Shervais (1980); (16) Stähle et al. (1990), Lu et al. (1997), Oppizzi and Schaltegger (1999), Schaltegger et al. (2008; 2011), Zanetti et al. (2013); (17) Bussy et al. (1998), Manzotti et al. (2018), Monjoie et al. (2007); (18) Kunz et al. (2018); (19) Bussy et al. (1998); (20) Burn (2016); (21) Rubatto (1998), Rubatto et al. (1999); (22) Bussy et al. (1998); Cenki-Tok et al. (2011); Paquette et al. (1989); Rubatto (1998) and Rubatto et al. (1999); (23) Kunz et al. (2018); (24) Rubatto (1998); (25) Babist et al. (2006); Cenki-Tok et al. (2011); Dal Piaz et al. (2001); Duchêne et al. (1997); Giuntoli et al. (2018a); Halama et al. (2014); Inger et al. (1996); Oberhänsli et al. (1985); Ramsbotham et al. (1994); Regis et al. (2014); Rubatto et al. (1999, 2011); Ruffet et al. (1997) and Venturini (1995)

The obtained age overlaps with the crystallization age of an Ivozio mafic rock of 355 ± 9 Ma reported by Rubatto (1998) and with the intrusion age of the metagabbro body of Cima di Bonze (350 ± 4 Ma, Rubatto et al. 1999). Carboniferous magmatism of a similar age has been reported in other portions of the Variscan chain, as extensively reported by Ballèvre et al. (2018) and Pohl et al. (2018). In the nearby Ivrea Zone, which also represents a portion of middle-to-lower Adriatic crust that escaped Alpine subduction, a crystallization age of 355 ± 6 Ma was obtained for an igneous felsic granulite (U–Pb in zircon, Vavra et al. 1996) (Fig. 11). In the Belledonne massif, U–Pb dating of zircon from trondhjemites gave an age of 367 ± 17 Ma (Ménot et al. 1988). In the internal part of the Briançonnais Zone, U–Pb dating of zircon from the Cogne diorite gave an age of 356 ± 3 (Bertrand et al. 2000; Guillot et al. 2012), while an older age of 371 ± 0.9 Ma was proposed by Bergomi et al. (2017). In the Bohemian massif, Sm–Nd whole-rock dating of gabbroic cumulates, diabase dikes and pillowed volcanics, resulted in an age of 351 ± 16 Ma (Pin et al. 1988). Similarly, the protolith of the Beja-Acebuches amphibolites and metagabbros in the Iberian Massif is dated at 332–340 Ma (zircon U–Pb, Azor et al. 2008).

The emplacement setting for the Carboniferous mafic rocks is not straightforward. The Bohemian Massif and the Beja-Acebuches are the only localities in the Variscan belt where the Carboniferous magmatism is attributed to an oceanic sequence. In the SZ, the Ivozio and Cima di Bonze gabbro bodies possibly intruded the continental basement during the Early Carboniferous (Rubatto 1998). However, the contacts between the metagabbros and the hosting metasediments are affected by the Alpine, and likely the early Permian tectonic evolution; therefore, any interpretation of those ages as related to the evolution of the SZ must use caution. The Ivozio metagabbro, similarly to what reported for the Cima di Bonze metagabbro (Rubatto 1998), does not show clear evidence for zircon growth during the early Permian as instead observed in the metasediments (see below). Mineralogical or geochronological evidence of a Permian metamorphism in the Ivozio metagabbro are lacking. We can only speculate that the structural position of the Ivozio gabbroic body during the Early Permian was such that this section escaped significant high-temperature (HT) metamorphism or that re-equilibration at HT conditions might have been limited by the lack of fluids.

### Permian metamorphism and magmatism

The investigated metasediments from Malone Valley (blueschist AV16-44, micaschist AV16-45 and Lws-micaschist AV16-51), Chiusella Valley (Mn-rich quartzite VC10-04) and Monte Mucrone (micaschist AV17-07) contain zircon rims with an early Permian age (Fig. 11). These rims are typically weakly zoned or unzoned; their chemical composition is characterized by variable Th/U ratios (0.01–0.36), a moderately steep HREE pattern and negative Eu anomaly. Small zircon cores are often rounded, with variable and complex internal zoning; the few scattered ages obtained for these cores (~ 645 to 346 Ma) suggest a detrital nature. Therefore, the early Permian zircon rims are interpreted as metamorphic overgrowth. In the case of Mn-rich quartzite VC10-04, we can speculate that the occurrence of two Permian zircon generations reflects multiple metamorphic events at high-temperature/low-pressure conditions, the age of which cannot be resolved (e.g. Manzotti et al. 2012). In this sample, not all the zircon cores have been analysed, and thus the presence of a detrital component cannot be excluded.

The Permian zircon ages from the SZ metasediments are consistent with the ages obtained from other metasediments from the central part of the EMC (~ 296 to ~ 285 Ma, Kunz et al. 2018) (Fig. 11). In all those metasediments, the textures indicate limited zircon dissolution (detrital rounded cores are commonly preserved) and new growth during HT metamorphism. These ages were attributed to the regional late Paleozoic HT metamorphism recorded in zircon across various Adriatic units of the Western Alps (EMC and IIDK in the SZ, Mt. Emilius Klippe and Valpelline series, Kunz et al. 2018 and references therein).

In the samples from Malone Valley, the metamorphic zircon age of the metasediments (292.3 ± 11.0 Ma to 285.9 ± 2.9 Ma) overlaps with the age of the zircon in the eclogite AV16-47 (282.0 ± 4.0 Ma). The age of the zircon in the mafic rock is interpreted as magmatic due to the steep REE pattern and the internal sector and fir-tree zoning. In the Monte Mucrone eclogite AV17-16 zircon cores show a variably steep M- to HREE pattern and a weak negative Eu anomaly, similar to REE patterns described for magmatic zircon in metagabbros from the Lanzo massif (Kaczmarek et al. 2008). They are therefore interpreted as magmatic with a crystallization age of 271 ± 10 Ma, which is slightly younger than the first metamorphic rim of the associated metasediment AV17-07 (289.4 ± 4.5 Ma). However, the eclogite crystallization age is calculated on only few (n = 4) data associated to a large uncertainty. The age of zircon metamorphic rim1 in sample AV17-07 corresponds to the intrusion age of the granitic body of Monte Mucrone, dated at ~ 297–285 Ma (Paquette et al. 1989; Bussy et al. 1998; Rubatto et al. 1999; Cenki-Tok et al. 2011). Magmatic zircon cores from a similar eclogitic boudin within the Monte Mucrone metasediments yielded and age of 285 ± 7 Ma (Rubatto et al. 1999), which overlaps with the granite intrusion age and is within uncertainty of our estimate. These samples are fully recrystallized under eclogite-facies conditions and thus the age of the relict magmatic zircon should be considered a minimum, as Pb loss during Alpine metamorphism cannot be excluded. Consequently, scattered dates significantly younger than the average ages are interpreted as resulting from Pb loss during a later metasomatic or metamorphic event.

The early Permian magmatic and metamorphic ages in the investigated samples are coeval with the bimodal Permian magmatism at ~ 295–280 Ma recorded all across the Western Alps (Paquette et al. 1989; Bussy et al. 1998; Rubatto et al. 1999; Monjoie et al. 2007; Cenki-Tok et al. 2011; Bergomi et al. 2017; Ballèvre et al. 2018; Manzotti et al. 2018) and with the age of the Mafic Complex formation in the Ivrea Zone (zircon in diorite: 285 + 7/− 5 Ma, Pin 1986; zircon in gabbro: 288 ± 4 Ma, Peressini et al. 2007; zircon in felsic and mafic intrusive: 282–286 Ma, Karakas et al. 2019) (Fig. 11). We conclude that contemporaneous Permian metamorphism and bimodal magmatism occurred in the SZ, similarly to what proposed for the Dent Blanche Tectonic System (Manzotti et al. 2018) and the Ivrea Zone (Ewing et al. 2013; Guergouz et al. 2018) (Fig. 11). This indicates that early Permian magmatism and HT metamorphism are linked to the same stage of extensional tectonics (e.g. Ewing et al. 2015; Manzotti et al. 2018) with high thermal regime that pervasively affected the South-Alpine and Austroalpine basements (e.g. Voshage et al. 1987; Vavra et al. 1996; Vavra and Schaltegger 1999; Mayer et al. 2000; Schuster and Stüwe 2008; Petri et al. 2017; Kunz et al. 2018), the Dent Blanche Tectonic System (e.g. Manzotti et al. 2012, 2018; Kunz et al. 2018) and the Briançonnais basement (Ballèvre et al. 2018). This event may be related either to the collapse of the Variscan belt or represents a separate tectonometamorphic cycle from the Variscan orogeny (e.g. Ballèvre et al. 2018), as already identified in the Eastern Alps (Schuster and Stüwe 2008).

The allanite from the blueschist AV16-44 from Malone Valley returns an age of 241.1 ± 6.2 Ma. There is little constraint on the conditions of allanite formation in this sample. No mineralogical relicts prior to Alpine metamorphism are present in the sample, apart from garnet cores and zircon grains, both interpreted as associated to the early Permian HT metamorphism. The small dated allanite crystals do not contain primary inclusions. The flat REE patterns of the dated allanite (Fig. 5) are distinct from those of magmatic allanite (Gregory et al. 2009) or of metamorphic allanite growing at HP conditions (quartz-rich micaschist, Regis et al. 2014). There is a similarity between the composition of allanite in our sample with that of allanite equilibrated at amphibolite to greenschist facies in metasediments from the Central Alps (calcschist, Boston et al. 2017), but the effect of different bulk rock compositions cannot be quantified. Therefore, we suggest that this allanite is not associated to a regional magmatic event and could reflect a metasomatic/metamorphic event related to the high geothermal gradient during Permo-Triassic extension.

The occurrence of scattered, < 270 Ma dates in zircon from the EMC has been previously described in the Monte Mucrone area (Rubatto 1998) and in the Lys Valley area (Kunz et al. 2018) in the central EMC. The observed textures related to fluid-assisted recrystallization (i.e. complete resetting of the chemical/isotopic system occurring at subsolidus conditions, Rubatto 2017) and U, Th and radiogenic Pb depletion in the Permian zircon grains have been attributed to late Permian and Triassic metasomatic stages (266 ± 4 Ma and 221 ± 14 Ma; Rubatto 1998; Kunz et al. 2018). Based on the available geochronological data, it is possible that multiple metasomatic events affected the SZ during the late Permian to Triassic extension in a setting with high geothermal gradients and thus favourable to mineral recrystallization. Post 270 Ma zircon ages attributed to metasomatic events have also been reported for the Ivrea Zone (e.g. Vavra et al. 1996; Vavra and Schaltegger 1999). In the Ivrea Zone, sporadic intrusions are dated in the Triassic (granitic dikes at 251 ± 2 Ma, Wright and Shervais 1980; the Finero Mafic Complex at 232 ± 3 Ma, Zanetti et al. 2013; and syenite dikes 225 ± 13 Ma, Stähle et al. 1990), alkaline pegmatoids at the Triassic-Jurassic boundary (210–190 Ma, Oppizzi and Schaltegger 1999; Schaltegger et al. 2008; 2011) and carbonate rocks related to sodic alkaline intrusions and amphibole mantle peridotites in the early Jurassic (187 ± 2.4 and 192 ± 2.5 Ma, Galli et al. 2019). This further supports a high geothermal gradient and circulation of fluids in the middle to lower crust of the Sesia and Ivrea zones between ~ 250 and 190 Ma, also enhanced by the intense deformation affecting these domains in the Triassic and the Jurassic (e.g. Vavra and Schaltegger 1999; Ewing et al. 2013, 2015).

### Alpine HP metamorphism

Among all the investigated samples, Alpine zircon rims were only found in the micaschist AV17-07 from Monte Mucrone (Figs. 10, 11). They are characterized by a relatively flat HREE distribution, lower HREE content compared to the other rim analyses and absence of Eu anomaly, which is a typical pattern for HP zircon in eclogites (Rubatto 2017 and references therein). The age of these zircon rims (67.4 ± 1.9 Ma) is therefore interpreted as dating eclogite-facies metamorphism. Ti-in-zircon thermometry of the Alpine rims gives temperatures of 575–615 °C, in line with the peak temperature proposed for this area (520–600 °C, Zucali et al. 2002).

Metamorphic zircon formation in sub-solidus conditions is not uncommon in HP rocks, but certainly not ubiquitous. Although restricted zircon net growth is expected at HP from a mass-balance point of view (Kohn et al. 2015), its formation can be generally ascribed to dissolution–precipitation enhanced by alkaline fluids (e.g. Rubatto 2017) and thus may be locally controlled. Indeed in the SZ the presence of Alpine zircon rims occurs only locally (i.e. in the central part of the EMC), and it is mainly documented in metasediments (Rubatto et al. 1999, 2011; Regis et al. 2014; Giuntoli et al. 2018a). According to petrological studies, the central portion of the EMC is the area that experienced the highest T conditions during Alpine subduction (up to 650 ± 50 °C, Giuntoli et al. 2018b), while the southern EMC reached maximum T of 500 °C (e.g. Pognante 1989a, b). Moreover, in the central portion of the EMC, the occurrence of multiple fluid pulses at HP has been inferred based on petrography and major element composition of HP metamorphic minerals such as garnet, phengite and amphibole (Konrad-Schmolke et al. 2011; Giuntoli et al. 2018b), while this is not the case in the southern EMC, where the main hydration stage occurred prior to or in the very early stage of subduction (Vho et al. 2020). While the solubility of Zr in aqueous fluids is low, it increases with increasing Si contents and alkalinity of the fluids (Ayers et al. 2012), both of which rise significantly with pressure (Hermann and Rubatto 2014; Rubatto 2017). The combination of slightly higher T conditions and more fluid circulation at HP in the central EMC with respect to the southern EMC might explain the paucity of HP zircon in the latter, compared to the central part of the EMC. In the area of Monte Mucrone, circulation of Si- and alkali-rich fluids at HP has been proposed to explain peculiar atoll and mushroom garnet textures (Robyr et al. 2013) and phengite veins in mafic eclogites (Vho et al. 2020), and such fluids might have locally enhanced zircon dissolution and recrystallization in this area. The 67.4 ± 1.9 Ma age for the HP zircon rims from Monte Mucrone overlaps within error with the eclogite-facies age of zircon rims in an eclogitic boudin from the same area (65 ± 5 Ma, Rubatto et al. 1999). Some of the Rb–Sr ages of white mica by Inger et al. (1996) in samples from Monte Mucrone and the nearby Mont Mars are also at the Cretaceous-Paleocene boundary (Fig. 1). In Monte Mucrone, Rb–Sr and Ar–Ar age analysis on single minerals and whole rock by Oberhänsli et al. (1985) range from 62 ± 3 Ma to 85 ± 1 Ma, while Th-Pb single dates of allanite by Cenki-Tok et al. (2011) range between 66 ± 3 Ma and 88 ± 1 Ma. Older Cretaceous HP ages of 75–85 Ma are also recorded in the central EMC by zircon and allanite (Rubatto et al. 2011; Regis et al. 2014) together with ~ 65 Ma ages. In the Cima di Bonze area (Fondo slice according to the definition of Regis et al. 2014), it has been proposed that a protracted involvement of parts of the Sesia crust in the subduction system led to two distinct HP stages (Yo-Yo subduction, Rubatto et al. 2011); it remains to be clarified if whether or not this is also the case for the Monte Mucrone rocks.

In the southern EMC, constraints on the age of the HP metamorphism are scarce and mostly obtained by phengite Rb–Sr dating (Fig. 1). No Cretaceous dates were found in zircon from this area as well as in the Ivozio Complex. In the case where Alpine zircon rims are rare, allanite can assist in constraining the time of the HP metamorphism (e.g. Rubatto et al. 2011; Regis et al. 2014). In the Malone Valley micaschist AV16-45, allanite is present in the main foliation associated with phengite (Fig. 4), but the high content of initial common Pb prevented the age from being constrained. Further investigation is necessary to better constrain the timing of the HP metamorphism in this area.

Alpine allanite was dated in the blueschist AV16-53 and in the eclogite AV16-57 from Ivozio Complex. In both samples, allanite coexists with glaucophane. In sample AV16-53 glaucophane grains are interpreted to have grown in equilibrium with garnet, whereas in sample AV16-57 textural observations indicate that glaucophane and phengite post-date the static growth of omphacite and garnet (see above). Clinopyroxene relics in sample AV16-53 are interpreted as pre-Alpine remnants and the glaucophane as part of the prograde-to-peak mineral assemblage together with garnet and zoisite (1.2–1.8 GPa and 450–550 °C, Zucali and Spalla 2011). The allanite age of 62.9 ± 4.2 Ma is therefore interpreted as dating a prograde stage close to the peak conditions (1.8 GPa, 520–600 °C). This age overlaps with the zircon age of 65 ± 3 Ma obtained in the surrounding micaschists by Rubatto et al. (1999) and interpreted as the HP peak age. In sample AV16-57, omphacite and garnet form the peak assemblage, and amphibole and phengite form at post-peak, but still HP, conditions (1.5–1.8 GPa, 500–600 °C, Zucali and Spalla 2011). The allanite age of 55.3 ± 7.3 Ma is associated to such post-peak stage, likely characterized by ingress of fluid in the eclogite as suggested by the presence of localised glaucophane + phengite pods and veins in which allanite was found. Fluids can mobilize significant amount of Pb; this might explain the higher common Pb content in allanite from sample AV16-57 with respect to that from sample AV16-53 (Table 5). The ages of the prograde metamorphism and of the incipient retrograde stage are not resolvable, but they assist in constraining the timing of the metamorphism of the Ivozio Complex. In the same area, an age of 76 ± 1 Ma was obtained by U–Pb zircon dating in a vein (Rubatto et al. 1999) and was attributed to a low-pressure stage. A more complete dataset would be needed to confirm this hypothesis and to clarify if those zircon grains crystallized during the prograde path, as proposed by Rubatto et al. (1999), or if the Ivozio Complex recorded a more complex subduction history characterized by two subsequent HP peaks as described for Cima di Bonze (Rubatto et al. 2011; Regis et al. 2014).

## Conclusions

In the Sesia Zone, three distinct magmatic and metamorphic events have been recorded and their age can be retrieved by in situ dating of zircon and allanite grains from various rock types.

1. The Ivozio mafic Complex intruded at 340.7 ± 6.8 Ma as part of an early Carboniferous magmatic phase that is also recorded by the Cima di Bonze gabbro, in the Ivrea Zone and in other portions of the Variscan belt. In the Ivozio and Cima di Bonze metagabbros, mineralogical or geochronological evidence of a Permian metamorphism are lacking, while they have been pervasively overprinted at HP conditions during Alpine subduction.
2. Early Permian magmatism and HT metamorphism are extensively recorded across the Sesia Zone. Combined with previous data, our results suggest that magmatism and metamorphism occurred roughly simultaneously. This concurs with similar scenarios proposed for other closely related tectonic units, e.g. the Ivrea Zone and the Dent Blanche Tectonic System, indicating that a high-thermal regime with associated magmatism broadly affected this portion of the future Adriatic continental margin during the early Permian.
3. Late Cretaceous to Paleocene HP metamorphism is associated to Alpine subduction in the Sesia Zone. In the EMC, mineral assemblages are intensively re-equilibrated at HP conditions, but this event is rarely recorded in zircon. Zircon rims yielding Alpine ages were found only in one sample from Monte Mucrone in the central portion of the EMC, while they are lacking in the southern EMC. It is speculated that zircon dissolution and growth was locally enhanced by the combination of slightly higher T conditions and more pervasive fluid circulation at HP in the central EMC with respect to the southern EMC. This would also justify the restricted distribution of published Alpine zircon ages, limited to the central area of the EMC, as due to a paucity of Alpine metamorphic rims in zircon grains from other localities. In the absence of Alpine zircon rims, dating of allanite (55.3 ± 7.3 Ma, 62.9 ± 4.2 Ma, Ivozio Complex) confirms metamorphism at the Cretaceous-Paleocene boundary.

## Supplementary information

**Additional file 1.** Microphotographs of samples from the different localities.

**Additional file 2.** Instrumental setup and operating conditions for LA-ICP-MS trace element analysis and dating.

**Additional file 3.** Weighted averages of zircon dates.

**Additional file 4.** Tables of LA-ICP-MS analyses of zircon from individual samples.

**Additional file 5.** Probability density plot for U-Pb zircon dates for individual samples and Ti-in-zircon temperatures for metamorphic rims in metasediments.

## Data Availability

All data generated or analysed during the current study are included in this published article and its additional files.
