# Supplementary material for: The evolution of the Sesia Zone (Western Alps) from Carboniferous to Cretaceous: insights from zircon and allanite geochronology
Source: Swiss J Geosci. 2020 Dec 7;113(1):24. doi: 10.1186/s00015-020-00372-4 (PMC7721683; doi:10.1186/s00015-020-00372-4)
Supplement: Supplementary file 1 — Additional file 1. Microphotographs of samples from the different localities. [file 15_2020_372_MOESM1_ESM.pdf]

The evolution of the Sesia Zone (Western Alps) from Carboniferous to Cretaceous: insights from zircon and allanite geochronology

Alice Vho, Daniela Rubatto, Pierre Lanari and Daniele Regis

Additional file 1

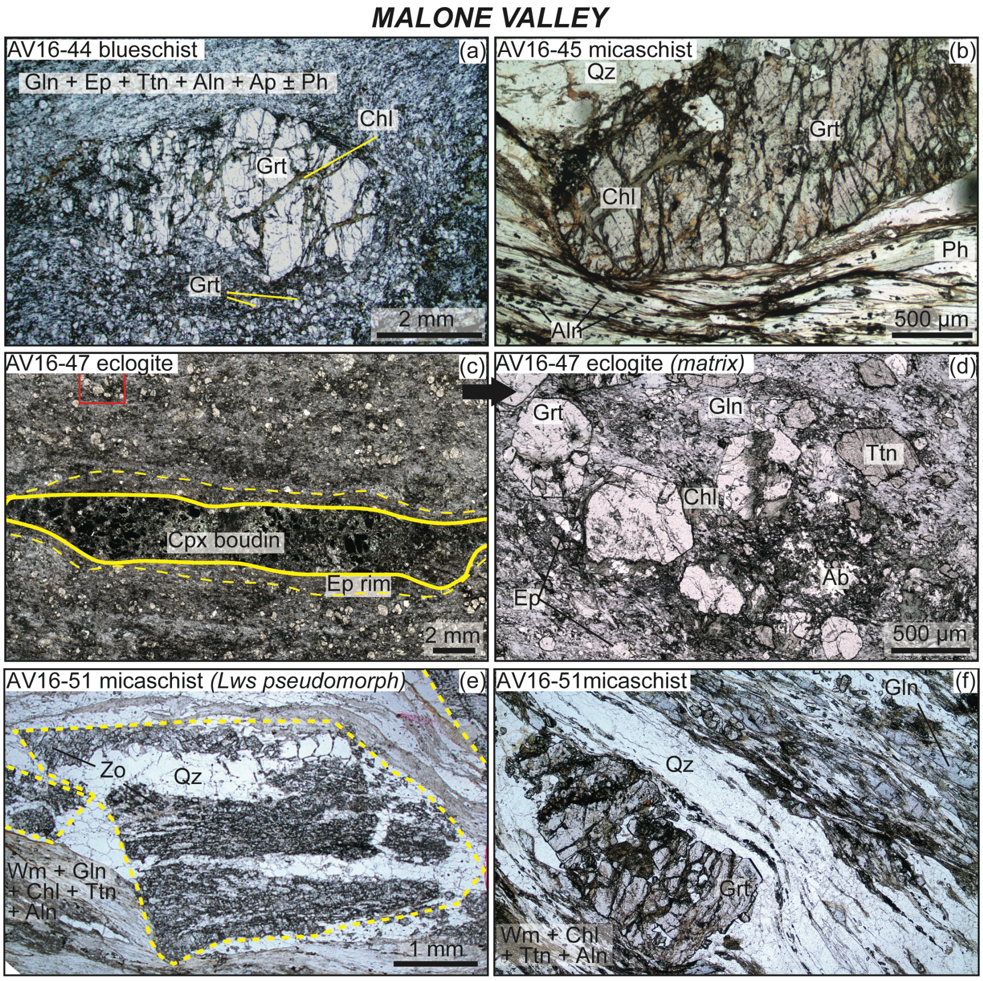

Figure AF1-1. Microphotographs of the samples from Malone Valley (parallel polarized light). (a) Blueschist AV16-44. A plurimillimeter porphyroblast is surrounded by the foliation marked by preferred orientation of glaucophane and epidote. Sub-millimetric euhedral garnet crystals are visible within the foliation. (b) Micaschist AV16-45: garnet porphyroblast surrounded by a foliation marked by phengite, chlorite, paragonite and allanite. (c,d) Eclogite AV16-47: boudin of clinopyroxenes with cloudy cores, rimmed by an epidote layer and surrounded by a weakly foliated matrix of garnet + glaucophane + titanite and retrograde albite and chlorite. (e,f) Micaschist AV16-51: lozenge-shape zoisite interpreted as pseudomorphs after lawsonite, wrapped by the main foliation (e), and garnet crystal surrounded by the foliation marked by preferred orientation of white mica, chlorite, glaucophane, titanite and allanite. Mineral abbreviations are from Whitney and Evans (2010).

IVOZIO COMPLEX

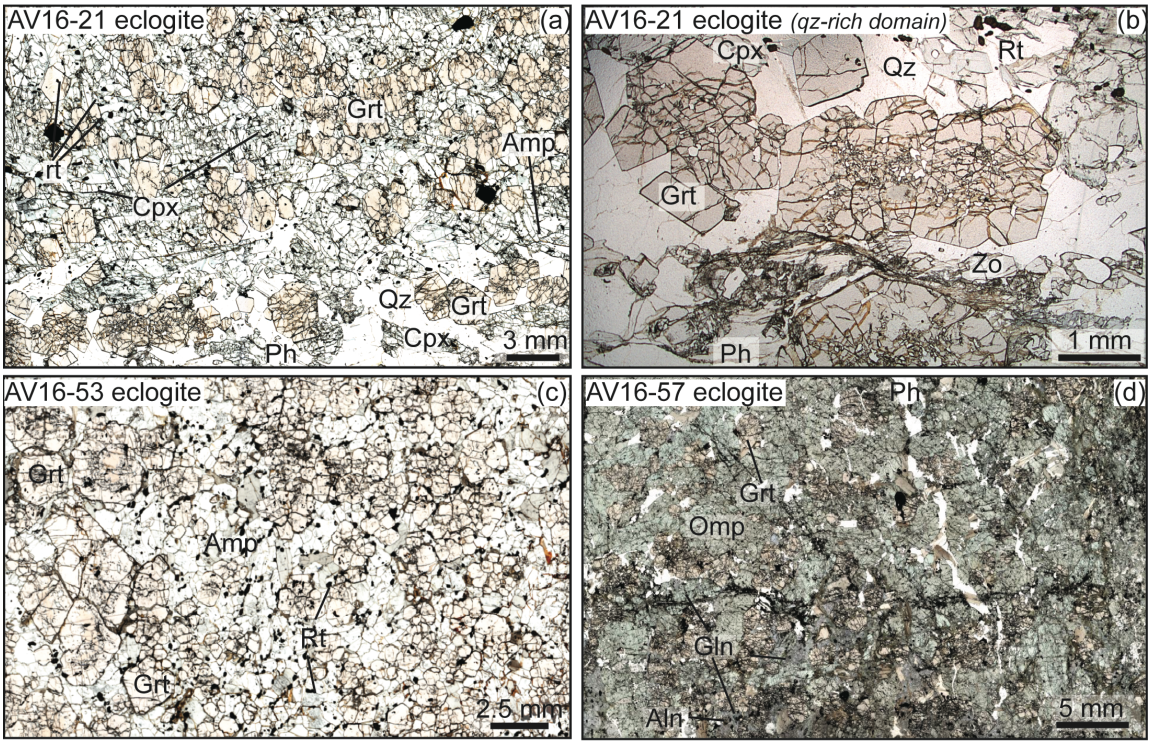

Figure AF1-2. Microphotographs of the samples from Ivozio Complex (parallel polarized light). (a) Eclogite AV16-21 has two distinct domains (separated by a red dashed line): a garnet + omphacite + rutile domain (upper part of the picture) and a garnet + quartz + phengite + amphibole domain (lower part). (b) Zoom in the quartz-rich domain; garnet shows an inclusion-rich core and a bright rim. (c) Blueschist AV16-53. (d) Eclogite AV16-57: glaucophane is absent in the upper part of the image, while it is present in the lower part of the image, where also allanite was found. Mineral abbreviations are from Whitney and Evans (2010).

CHIUSELLA VALLEY

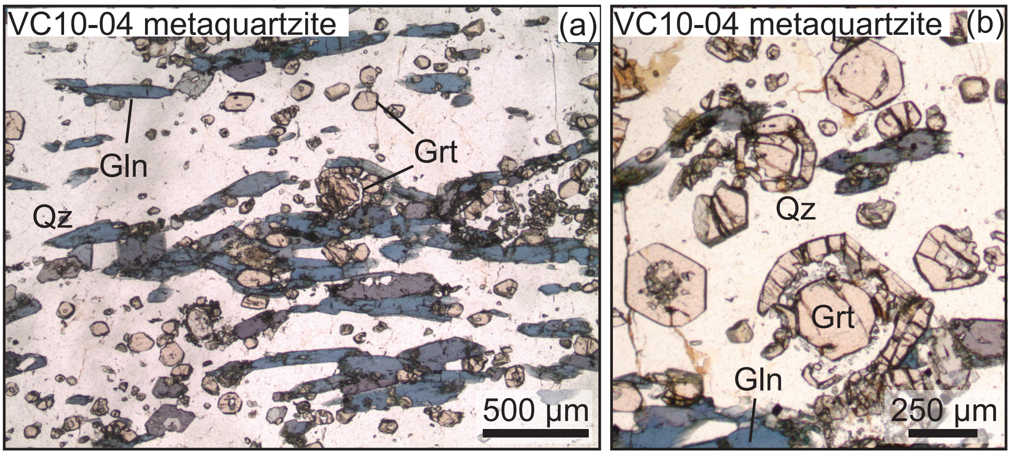

Figure AF1-3. Microphotographs of sample VC10-04 from Chiusella Valley (parallel polarized light). (a,b) Mn-rich metaquartzite VC10-04 containing elongated glaucophane blasts with a preferred orientation and parallel quartz and garnet layers. Details of the garnet atoll texture are shown in (b). Mineral abbreviations are from Whitney and Evans (2010).

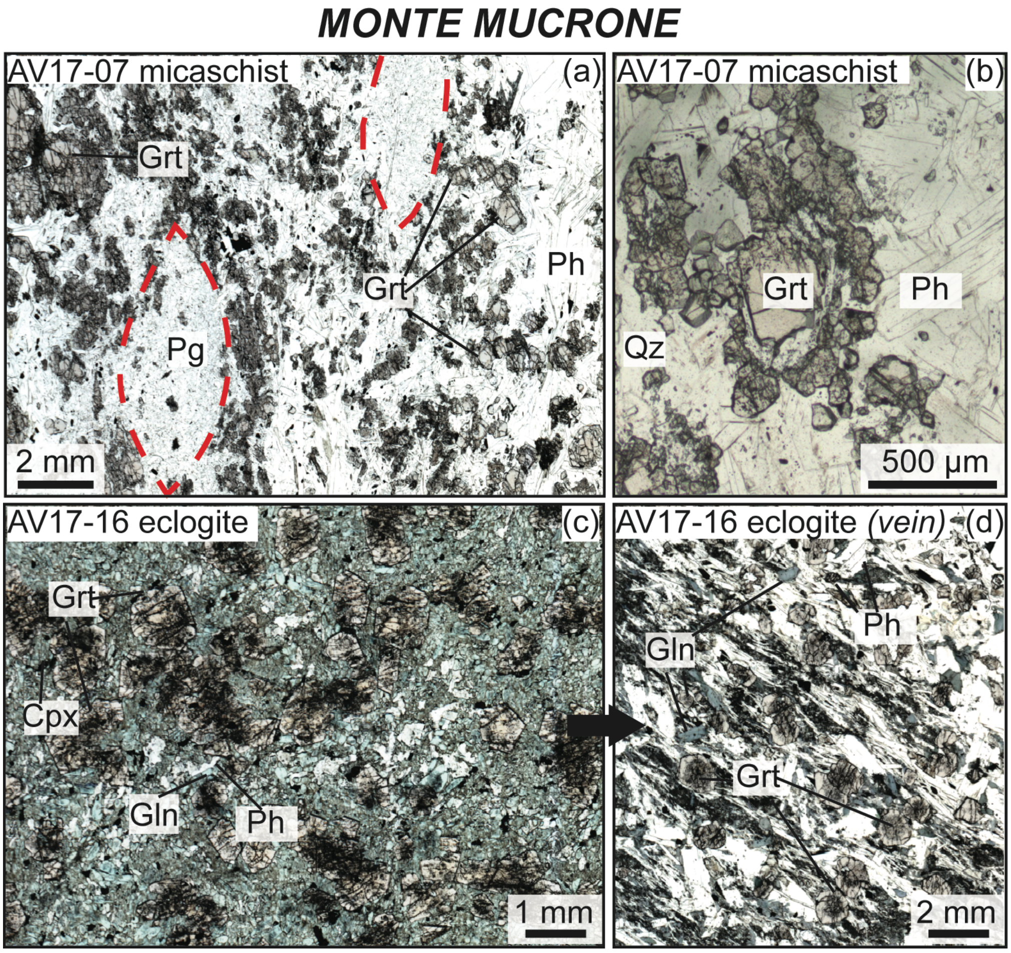

Figure AF1-4. Microphotographs of samples from Monte Mucrone (parallel polarized light). (a,b) Micaschist AV17-07 containing large phengite grains and lozenge-shape aggregates of fine-grained paragonite. Garnet consists of millimetre porphyroclastic cores surrounded by euhedral smaller grains (detail shown in (b)) and as submillimetre euhedral grains with quartz inclusion distribution between core and rim (forming atoll garnet). Mineral abbreviations are from Whitney and Evans (2010).
