# Supplementary material for: The evolution of the Sesia Zone (Western Alps) from Carboniferous to Cretaceous: insights from zircon and allanite geochronology
Source: Swiss J Geosci. 2020 Dec 7;113(1):24. doi: 10.1186/s00015-020-00372-4 (PMC7721683; doi:10.1186/s00015-020-00372-4)
Supplement: Supplementary file 2 — Additional file 2. Instrumental setup and operating conditions for LA-ICP-MS trace element analysis and dating. [file 15_2020_372_MOESM2_ESM.pdf]

The evolution of the Sesia Zone (Western Alps) from Carboniferous to Cretaceous: insights from zircon and allanite geochronology

Alice Vho, Daniela Rubatto, Pierre Lanari and Daniele Regis

Additional file 2

Table AF2-T1. LA-ICP-MS trace element analysis instrumental setup and operating conditions (zircon in samples AV16-47, AV16-21; allanite in sample AV16-44).

**Laboratoryand Sample Preparation**	
Laboratory name	Department of Geological Sciences, University of Bern
Sample type/mineral	Zircon
Sample preparation	Conventional mineral separation, 1 inch resin mount, 1 um polish to finish
Imaging	Contrast Charge images, ZEISS EV050, low vacuum conditions (18 Pa), 12 kV, ca. 1 nA, working distance ca. 10 mm
**Laser ablation system**	
Make, Model and type	GeoLasPro (Compex 102; Lambda Physics)
Ablation cell and volume	In-house built rectangle cell brought down to a round base area, volume ca. 21 cm^3
Laser wavelength	193 nm ArF excimer laser
Fluence	2.5 $J{\bullet}cm^{-2}$
Repetition rate	9 Hz
Spot size	24 µm, round
Sampling mode / pattern	Single spot
Carrier gas	He (1.000 $L{\bullet}min^{-1}$) and H_2 (0.008 $L{\bullet}min^{-1}$) cell gas fow, Ar make-up gas combined using a Y-piece 50% along the sample transport line to the torch
Background collection	40 s
Ablation duration	40 s
Wash-out delay	20 s
Cell carrier gas flow	1.008 $L{\bullet}min^{-1}$
**ICP-MS Instrument**	
Make, Model and type	Elan DRC-e (Perkin Elmer) ICP-QMS
Sample introduction	Via conventional tubing
RF power	1550 W
Make-up gas flow	0.65 – 0.70 $L{\bullet}min^{-1}$ Ar
Detection system	Dual (cross-calibrated pulse/analog modes)
Integration time per peak	10 to 20 ms
**Data Processing**	
Gas blank	30 seconds on-peak
Calibration strategy	NIST-612 (zircon), NIST-610 (allanite) glasses as primary reference material<br>BCR-2G (zircon), NIST-612 (allanite) glasses as secondary reference material
Reference Material info	NIST-610, NIST-612 (Jochum et al. 2011)<br>BCR-2G (Jochum et al. 2005)
Data processing package	SILLS (Guillong et al., 2008)
Mass discrimination	Standard-sample bracketing normalized to reference material
Internal standardization	Si wt% 14.86 (zircon), Si wt% 31.00 (allanite)
Accuracy and precision	BCR-2g concentrations within 10% of nominal value, Yb within 15%<br>NIST-612 concentrations within 3% of nominal value<br>SD on repeated analyses typically within 5 %

Table AF2-T2. LA-ICP-MS trace element analysis instrumental setup and operating conditions (samples AV16-45, AV16-51, AV17-07, AV17-16).

**Laboratoryand Sample Preparation**	
Laboratory name	Insititut für Geologie, Universität Bern
Sample type/mineral	Zircon
Sample preparation	Conventional mineral separation, 1 inch resin mount, 1 um polish to finish
Imaging	Contrast Charge images, ZEISS EV050, low vacuum conditions (18 Pa), 12 kV, 1 nA, working distance of ca. 10 mm
**Laser ablation system**	
Make, Model and type	RESOlution Laser System SE, ATL Laser head
Ablation cell and volume	Laurin Technic S155 Cell, two volumes cell, volume ca. 1-2 cm^3
Laser wavelength	193 nm
Fluence	4 $J\bullet cm^{-2}$
Repetition rate	5 Hz
Spot size	20 µm, round
Sampling mode / pattern	Single spot
Carrier gas	100% He, Ar make-up gas (450 ml/min) and N_2 (3ml/min) combined using the Squid® device from RESOlution Instruments.
Background collection	30 secs
Ablation duration	40 secs
Wash-out delay	20 secs
Cell carrier gas flow	0.75 l/min
**ICP-MS Instrument**	
Make, Model and type	Agilent 7900, Q-ICP-MS
Sample introduction	Via conventional tubing
RF power	1300 W
Make-up gas flow	0.87 l/min Ar
Detection system	Single collector secondary electron multiplier
Integration time per peak	8 to 30 ms
**Data Processing**	
Gas blank	30 seconds on-peak
Calibration strategy	NIST-612 glass as primary reference material,<br>GSD-1g glass as secondary reference material
Reference Material info	NIST-612 (Jochum et al. 2011)<br>GSD-1g (Jochum et al. 2005)
Data processing package	IOLITE (Paton et al. 2011)
Mass discrimination	Standard-sample bracketing normalized to reference material
Internal standardization	Si wt% 14.86
Accuracy and precision	GSD-1g concentrations within 15% of nominal value<br>STDEV on repeated analysis within 2 %

Table AF2-T3. LA-ICP-MS allanite dating instrumental setup and operating conditions.

**Laboratoryand Sample Preparation**	
Laboratory name	Department of Geological Sciences, University of Bern
Sample type/mineral	Allanite
Sample preparation	Thin section or conventional mineral separation, 1 inch resin mount, 1 um polish to finish
Imaging	Contrast Charge images, ZEISS EV050, low vacuum conditions (18 Pa), 18 kV, ca. 1 nA, working distance ca. 10 mm
**Laser ablation system**	
Make, Model and type	GeoLasPro (Compex 102; Lambda Physics)
Ablation cell and volume	In-house built rectangle cell brought down to a round base area, volume ca. 21 cm^3
Laser wavelength	193 nm ArF excimer laser
Fluence	2.5 J•cm^{-2}
Repetition rate	9 Hz
Spot size	24 µm, round
Sampling mode / pattern	Static spot ablation
Carrier gas	He (1.000 L•min^{-1}) and H_2 (0.008 L•min^{-1}) cell gas fow, Ar make-up gas combined using a Y-piece 50% along the sample transport line to the torch
Background collection	60 s
Ablation duration	40 s
Wash-out delay	15 s
Cell carrier gas flow	1.008 L•min^{-1}
**ICP-MS Instrument**	
Make, Model and type	Elan DRC-e (Perkin Elmer) ICP-QMS
Sample introduction	Via conventional tubing
RF power	1550 W
Make-up gas flow	0.65 – 0.70 L•min^{-1} Ar
Detection system	Dual (cross-calibrated pulse/analog modes)
Integration time per peak	10 to 30 ms
Masses measured	27, 29, 202, 204, 206, 207, 208, 232, 235, 238
Total integration time per output data point (s)	Dwell time: 180 ms $sweep^{-1}$<br>222 sweeps acquired per spot
IC dead time (ns)	55 ns
**Data Processing**	
Gas blank	60 s on-peak zero subtracted
Calibration strategy	Plešovice zircon as primary standard<br>CAP and SISS allanite as secondary standard
Reference Material info	Plešovice U/Pb age = 337.1 ± 0.4 Ma (Sláma et al. 2008)<br>CAP $^{232}Th/^{208}Pb$ age = 275 ± 2 Ma (Barth et al. 1994)<br>SISS $^{232}Th/^{208}Pb$ age = 31.5 ± 0.5 Ma (von Blackenburg 1992)
Data processing package	TRINITY (Burn et al. 2017)
Mass discrimination	Standard-sample bracketing normalized to reference material
Accuracy and precision	Secondary standard $^{232}Th/^{208}Pb$ age for each analytical block (in Ma, ± 2σ):<br>CAP (sample AV16-44): 277 ± 5; 280 ± 3; 282 ± 7<br>SISS (sample AV16-57): 30.9 ± 0.8; 30.7 ± 0.7<br>SISS (sample AV16-45): 31.1 ± 1.0; 30.0 ± 1.2; 29.5 ± 1.2; 29.0 ± 1.7<br>SISS (sample AV16-53): 29.9 ± 0.7; 29.9 ± 1.3; 30.0 ± 1.6
Uncertainty level and propagation	Ages are quoted at 95% confidence level. Age uncertainty of reference material and common-Pb composition uncertainty are propagated where appropriate (see Burn et al. 2017 for details).
