# Supplementary material for: The evolution of the Sesia Zone (Western Alps) from Carboniferous to Cretaceous: insights from zircon and allanite geochronology
Source: Swiss J Geosci. 2020 Dec 7;113(1):24. doi: 10.1186/s00015-020-00372-4 (PMC7721683; doi:10.1186/s00015-020-00372-4)

The evolution of the Sesia Zone (Western Alps) from Carboniferous to Cretaceous: insights from zircon and allanite geochronology

Alice Vho, Daniela Rubatto, Pierre Lanari and Daniele Regis

Additional file 3 - Weighted averages of zircon dates.

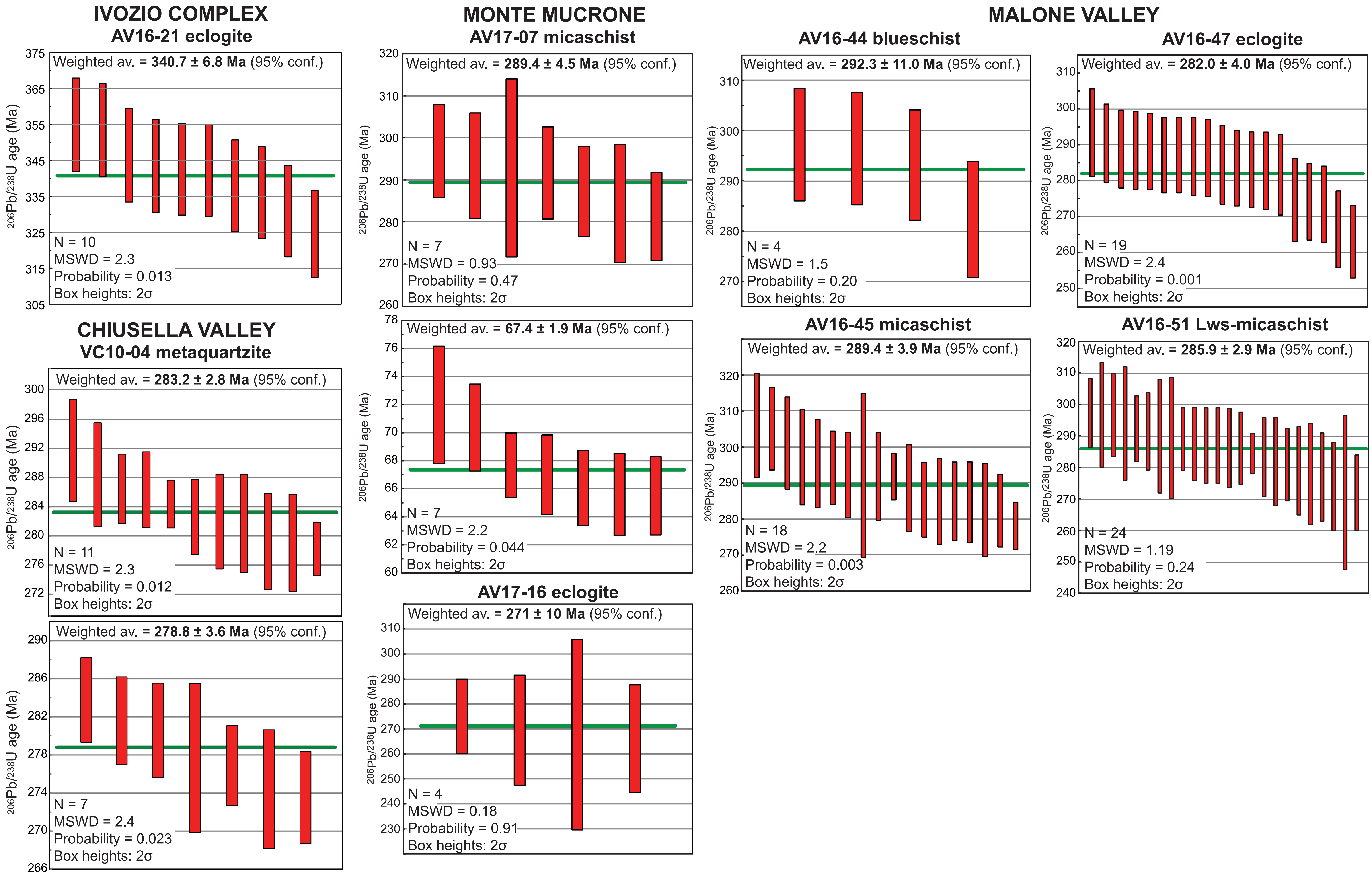

Supplement: Supplementary file 3 — Additional file 3. Weighted averages of zircon dates. [file 15_2020_372_MOESM3_ESM.pdf]
