# Supplementary material for: The evolution of the Sesia Zone (Western Alps) from Carboniferous to Cretaceous: insights from zircon and allanite geochronology
Source: Swiss J Geosci. 2020 Dec 7;113(1):24. doi: 10.1186/s00015-020-00372-4 (PMC7721683; doi:10.1186/s00015-020-00372-4)

The evolution of the Sesia Zone (Western Alps) from Carboniferous to Cretaceous: insights from zircon and allanite geochronology

Alice Vho, Daniela Rubatto, Pierre Lanari and Daniele Regis

Additional file 5.

(a-d) Probability density plot for U-Pb zircon dates for individual samples from Malone Valley. In metasediments (AV16-44, AV16-45, AV16-51), only metamorphic rim dates are plotted. (e,f) Probability density plot for U-Pb zircon dates for individual samples from Monte Mucrone. In metasediment AV17-07, only metamorphic rim dates are plotted. This sample is the only one where a population of late Cretaceous age was measured. (g) Ti-in-zircon temperatures for zircon metamorphic rims in metasediments (calibration of Watson et al. 2006). Only temperatures below 850 °C were considered reliable and are reported (see text for details). Samples are colour-coded, and spots associated to Alpine ages and showing a typical HP REE pattern in sample AV17-07 are represented in green in (e) and (g).

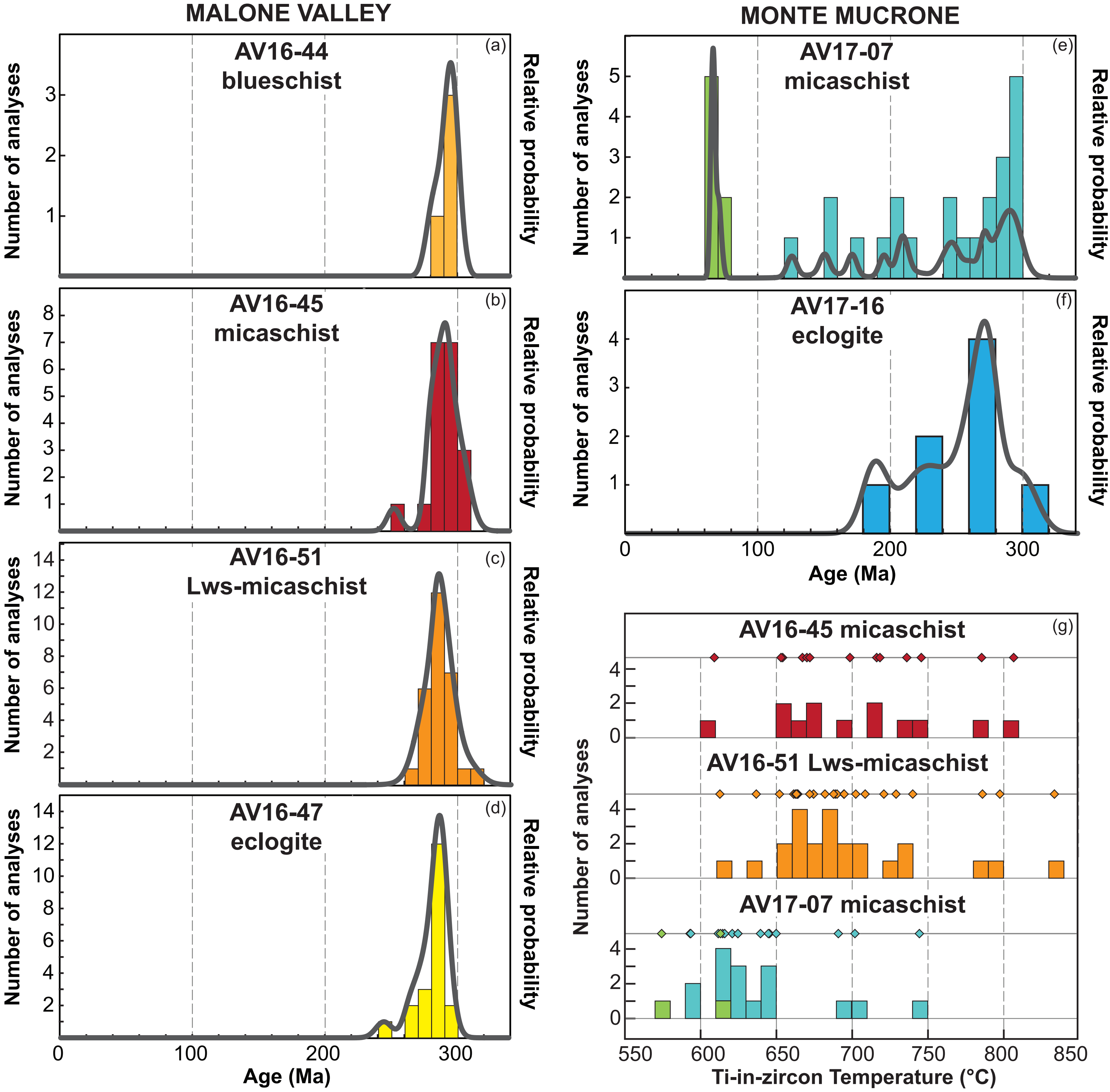

Supplement: Supplementary file 5 — Additional file 5. Probability density plot for U-Pb zircon dates for individual samples and Ti-in-zircon temperatures for metamorphic rims in metasediments. [file 15_2020_372_MOESM5_ESM.pdf]
